# Development of a Gold Nanoparticle-Based Lateral-Flow Immunoassay for *Pneumocystis* Pneumonia Serological Diagnosis at Point-of-Care

**DOI:** 10.3389/fmicb.2019.02917

**Published:** 2019-12-19

**Authors:** Ana Luísa Tomás, Miguel P. de Almeida, Fernando Cardoso, Mafalda Pinto, Eulália Pereira, Ricardo Franco, Olga Matos

**Affiliations:** ^1^Medical Parasitology Unit, Group of Opportunistic Protozoa/HIV and Other Protozoa, Global Health and Tropical Medicine, Instituto de Higiene e Medicina Tropical, Universidade NOVA de Lisboa, Lisbon, Portugal; ^2^UCIBIO, REQUIMTE, Departamento de Química, Faculdade de Ciências e Tecnologia, Universidade NOVA de Lisboa, Caparica, Portugal; ^3^REQUIMTE/LAQV, Departamento de Química e Bioquímica, Faculdade de Ciências da Universidade do Porto, Porto, Portugal

**Keywords:** *Pneumocystis* pneumonia, gold nanoparticles, point-of-care, lateral-flow immunoassay, serological diagnosis, major surface glycoprotein, kexin-like serine protease 1, synthetic recombinant antigens

## Abstract

*Pneumocystis jirovecii* pneumonia (PcP) is a major human immunodeficiency virus (HIV)-related illness, rising among immunocompromised non-HIV patients and in developing countries. Presently, the diagnosis requires respiratory specimens obtained through invasive and costly techniques that are difficult to perform in all patients or implement in all economic settings. Therefore, the development of a faster, cost-effective, non-invasive and field-friendly test to diagnose PcP would be a significant advance. In this study, recombinant synthetic antigens (RSA) of *P. jirovecii*’s major surface glycoprotein (Msg) and kexin-like serine protease (Kex1) were produced and purified. These RSA were applied as antigenic tools in immunoenzymatic assays for detection of specific anti-*P. jirovecii* antibodies (IgG and IgM) in sera of patients with (*n* = 48) and without (*n* = 28) PcP. Results showed that only IgM anti-*P. jirovecii* levels were significantly increased in patients with PcP compared with patients without *P. jirovecii* infection (*p* ≤ 0.001 with both RSA). Thus, two strip lateral flow immunoassays (LFIA), based on the detection of specific IgM anti-*P. jirovecii* antibodies in human sera samples, were developed using the innovative association of *P. jirovecii’*s RSA with spherical gold nanoparticles (AuNPs). For that, alkanethiol-functionalized spherical AuNPs with ca. ~40 nm in diameter were synthetized and conjugated with the two RSA (Msg or Kex1) produced. These AuNP-RSA conjugates were characterized by agarose gel electrophoresis (AGE) and optimized to improve their ability to interact specifically with serum IgM anti-*P. jirovecii* antibodies. Finally, two LFIA prototypes were developed and tested with pools of sera from patients with (positive sample) and without (negative sample) PcP. Both LFIA had the expected performance, namely, the presence of a test and control red colored lines with the positive sample, and only a control red colored line with the negative sample. These results provide valuable insights into the possibility of PcP serodiagnosis at point-of-care. The optimization, validation and implementation of this strip-based approach may help to reduce the high cost of medical diagnosis and subsequent treatment of PcP both in industrialized and low-income regions, helping to manage the disease all around the world.

## Introduction

The fungus *Pneumocystis jirovecii* is a pathogen able to cause a fatal pneumonia (PcP) in immunocompromised patients worldwide (Barry and Johnson, 2001; Huang et al., 2011; Esteves et al., 2014; Matos et al., 2017). In industrialized countries, the incidence of PcP has decreased with the widespread use of chemoprophylaxis and the introduction of combination antiretroviral therapy, but it still remains a serious clinical problem for human immunodeficiency virus (HIV)-infected patients (Huang et al., 2011; Esteves et al., 2014; Matos et al., 2017; European Centre for Disease Prevention and Control (ECDC)/WHO Regional Office for Europe, 2018). Likewise, the rising number of immunocompromised non-HIV-infected patients susceptible to *P. jirovecii* infection in these countries, warrants the need for improved diagnostic and treatment strategies (Hughes, 2005; Roux et al., 2014). In developing countries, where there is a lack of diagnostic resources and expertise, the number of PcP cases reported have been increasing significantly as more sensitive/specific laboratory methods are being used (Chakaya et al., 2003; van Oosterhout et al., 2007; Huang et al., 2011; Matos, 2012; Esteves et al., 2014; Morrow et al., 2014).

Despite all the advances in understanding *P. jirovecii* infection over the last years, in the twenty-first century the standard diagnosis of this disease still depends on the detection of *P. jirovecii* organisms through expensive and laborious technologies (cytochemical or immunofluorescent staining and/or PCR) applied to respiratory specimens obtained by invasive techniques, such as bronchoscopy (Alanio et al., 2016; Matos and Esteves, 2016; Matos et al., 2017; Tomás and Matos, 2018). These standard diagnosis methods, besides being difficult to implement in all economic settings, are not always possible to perform in patients with respiratory failure or in children (Alanio et al., 2016; Matos and Esteves, 2016; Matos et al., 2017; Tomás and Matos, 2018). Therefore, to improve disease management worldwide, there is a need to develop and implement an alternative approach for the diagnosis of PcP that can reduce associated costs, the need for invasive procedures, and also improves response time and specificity.

Lateral flow immunoassays (LFIA) offer an easy solution to these limitations as they are a simple, rapid and user friendly technique, that do not require time-consuming instrumental methods or technical expertise, allowing a low-cost point-of-care alternative (Chan et al., 2013; Pöhlmann et al., 2014; Li et al., 2015; Singh et al., 2015). Although LFIA is a well-recognized technique, a specific serological biomarker for PcP diagnosis has not been established (Morris and Masur, 2011; Esteves et al., 2015; Matos and Esteves, 2016). Yet, reports of protection against acquisition of infection by passive transfer of immune sera in mice (Gigliotti et al., 2002) and by vaccination in immunosuppressed non-human primates (Kling and Norris, 2016), triggers interest in serum antibodies as serological biomarkers of the disease. In addition, the suggestion that the IgM isotype has a predominant role in shaping the earliest steps in recognition and clearance of *Pneumocystis* infection both in mice (Rapaka et al., 2010) and in humans (Djawe et al., 2010; Tomás et al., 2016), not only support the role of antibodies in disease protection, but also highlights the idea that a serological test for PcP diagnosis is viable.

As *P. jirovecii*’s major surface glycoproteins (Msg) are characteristic of this microorganism and highly immunogenic, containing both B and T cell protective epitopes (Stringer and Keely, 2001), they are the obvious candidate to study serological responses. In fact, promising studies using recombinant antigens of this protein and antibody immunodetection techniques, have shown that patients with PcP or previous episodes of PcP present higher serum levels of anti-*P. jirovecii* antibodies than patients without *P. jirovecii* infection or without previous PcP events (Daly et al., 2004; Djawe et al., 2010; Gingo et al., 2011; Blount et al., 2012; Tomás et al., 2016). However, as Msg presents antigenic variation during infection as an evasion mechanism (Kling and Norris, 2016; Hauser, 2019), other antigenic candidates began to been explored. *Pneumocystis* kexin-like serine protease 1 (Kex1) is one of them, because it holds an antigenically stable active site peptide sequence coded by a nuclear single-copy gene (Kutty and Kovacs, 2003; Esteves et al., 2009), which avoids possible genetic variation. Therefore, recombinant Kex1 antigens were also used to study the humoral response to *P. jirovecii*, and the results suggest that a high humoral response to this protein can be detected and correlates with disease protection (Gingo et al., 2011; Kling and Norris, 2016).

Taking this into consideration and knowing that measuring the presence of biomarkers becomes quicker, more sensitive and more flexible when nanoparticles are put to work as tags or labels (Baptista et al., 2008; Baptista et al., 2011; Almeida et al., 2014), led to the idea to develop an immunonanodiagnostic platform for PcP diagnosis at point-of-care. Gold nanoparticles (AuNPs) are the nanomaterial most commonly used in the development of nanotechnology approaches for clinical diagnosis because of their ability to form conjugates with biomolecules (e.g., proteins and oligonucleotides) and due to their high surface area, stability and intense color (Nagatani et al., 2006; Baptista et al., 2008; Wilson, 2008; Franco and Pereira, 2013; O’Farrel, 2013; Almeida et al., 2014). Thus, in this study, a bionanodiagnostic platform for PcP diagnosis was developed associating recombinant synthetic antigens of *P. jirovecii*’s Msg and Kex1 with functionalized gold nanoparticles, in order to improve detection of specific anti-*P. jirovecii* antibodies in human sera samples.

**FIGURE 1:**
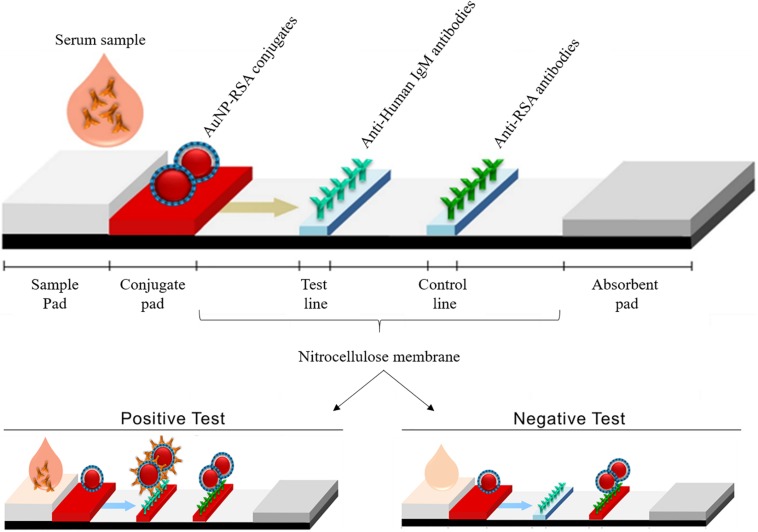
Illustration of LFIA strips developed in this study, showing its various components and the expected results in positive and negative tests. In a positive test, AuNP-RSA conjugates will bind to IgM anti-*P. jirovecii* antibodies present in the serum of infected patients, forming a complex that is captured by the immobilized anti-human IgM in the test line that becomes red colored. AuNP-RSA conjugates in excess will continue to migrate from the test line to the control line, where immobilized anti-RSA antibodies will capture them, giving rise to a second red line. In the negative test, as IgM anti-*P. jirovecii* antibodies are absent in the patient’s serum, no complex is formed with AuNP-RSA conjugates, which precludes the interaction with anti-human IgM antibodies, preventing the color formation at the test line (no color). In the control line, the AuNP-RSA conjugates will be captured by the immobilized anti-RSA antibodies, giving rise to a red line.

This platform, illustrated in Figure 1, was developed using AuNP-RSA conjugates to detect IgM anti-*P. jirovecii* antibodies in patients sera, reactive to either of the RSA, in order to allow less invasive biological specimens to be used in the diagnosis of this infectious disease. These LFIA prototypes intends to be specific, sensitive and accurate for PcP diagnosis, making a highly relevant contribution to public health and economy in industrialized countries and in communities with low-income and lack of technology, helping to manage the disease worldwide.

## Materials and Methods

### Production of *P. jirovecii’*s Recombinant Synthetic Antigens (RSA) and Anti-RSA Antibodies

#### RSA Production and Expression

The Msg RSA was designed as previously described (Tomás et al., 2016) and Kex1 RSA followed the same procedure, studying the immunogenicity of the *P. jirovecii*’s KEX1 longest gene sequences available (GenBank: AY130996.1 and AY127566.1) (Kutty and Kovacs, 2003). The putative reactive epitopes were selected using bioinformatic tools to analyze electrochemical properties, secondary structure prediction, polarity, relative position to the membrane and hydrophobicity profile of the specific polypeptides at the online software ExPASy – ProtScale and CBS – TMHMM – version 2.0 (Dai et al., 2012, 2013; Tomás et al., 2016). Only specific and conserved regions of *Pneumocystis* KEX1 genes, presenting high similarity with the sequences previously reported in GenBank for *Pneumocystis*’ Kex1 protein were considered for the final selection of the RSA composition, through the NCBI blast tool. Regions with high-predicted antigenicity and reactivity were chosen and DNA oligonucleotides coding these regions (connected by pentaglycine residues) were synthesized and cloned into the plasmid pHTP0 (Nzytech^®^). The insert sequences were confirmed by sequencing (Stabvida^®^). After synthesis, each RSA was cloned into the expression vector pLATE 31 (#K1261, Thermo Scientific^®^), following manufacturer’s instructions. This process enabled the synthetic production of both RSA with a polyhistidine tail end (6xHis), allowing their purification by immobilized metal ion affinity chromatography (IMAC). These vectors were used to clone CaCl_2_ competent *E. coli XJb (DE3)* cells by heat shock. Cell pellets from 15 mL of IPTG-induced cultures were re-suspended in 6 mL of lysis buffer (10 mM Tris-HCl, 50 mM KCl, 1 mM EDTA, 0.2 mM PMSF, 500 mM NaCl, 1% Triton X-100, 0.5% CHAPS, 2 mM CaCl_2_ and 0.02 μg.mL^–1^ DNAse I) and subjected to three cycles of a freeze-thaw method. The supernatants were removed and the pellets were re-suspended in 6 mL of 8 M Urea. An IMAC purification system (HiTrap columns, GE Healthcare^®^) packed with a histidine chelating resin (Ni Sepharose^TM^ 6 FastFlow, GE Healthcare^®^) was used. The column was balanced with 10 mL ligation buffer [20 mM Na_2_HPO_4_, 500 mM NaCl, 20 mM Imidazole (pH = 7.4)], then the sample containing the RSA was applied. The column was washed with 10 mL of ligation buffer, and the RSA were eluted with 20 mL of elution buffer [20 mM Na_2_HPO_4_, 500 mM NaCl, 500 mM Imidazole, 5% (v/v) glycerol (pH = 7.4)]. The eluted proteins were desalted with a desalting membrane (D-0655, Sigma-Aldrich^®^) into a new buffer [20 mM Na_2_HPO_4_, 20 mM NaCl, 2% (v/v) glycerol (pH = 7.4)], analyzed by sodium dodecyl sulfate-polyacrylamide gel electrophoresis (SDS-PAGE) on 15% acrylamide gels and by indirect ELISA using anti-polyhistidine antibodies. Their final concentrations were determined (NanoDrop 1000, Thermo Scientific^®^) before use.

#### Polyclonal Anti-RSA Antibodies Production

In order to produce antibodies able to recognize the RSA produced, ascitic fluids containing polyclonal anti-RSA antibodies were produced following the protocol of Ou et al. (1993), with modifications. Briefly, after RSA purification, 50 μg of each RSA were emulsified in incomplete Freund’s adjuvant plus peptide adjuvant and the emulsion was injected intraperitoneally into five 5–6 weeks old BALB/c male mice for each RSA. Subsequent immunizations with 50 μg of RSA emulsified in incomplete Freund’s adjuvant, took place at 2–3 weeks interval. After four inoculations, a test-bleed (0.5 mL per animal) was tested by ELISA for titration. When serum titers against the RSA were greater or equal to 1:1000, an intraperitoneal injection of 1 × 10^6^ cells of sarcoma 180 cell line was performed in each mouse, to obtain high titer polyclonal ascitic fluids. When a 20–25% increase in body weight of each mouse was recorded (caused by the ascite), they were euthanized and the ascitic fluids were collected and quantified (NanoDrop 1000, Thermo Scientific^®^). These fluids will be referred throughout this work as “AuNP-RSA antibodies.”

All animals used in the study were housed and cared for under the guidelines set forth in the Guide for the Care and Use of Laboratory Animals. Good animal handling practices were used and all European Directives were followed, namely the Portuguese DL 113/2013. Experiments were conducted by people certified by the Portuguese national body for experimental animal manipulations “Direcção Geral de Alimentação e Veterinária” (DGAV). The facilities at Instituto de Higiene e Medicina Tropical/Universidade Nova de Lisboa (IHMT/UNL) animal house, procedures for maintenance and care of animals as well the experimental scheme used (immunization, blood collection, spleen removal, and euthanasia procedures) are accredited by the Portuguese DGAV and in accordance with relevant guidelines and regulations of the ethical committee of IHMT/UNL.

### Human Serum Specimens

This study retrospectively analyzed sera from 76 HIV-infected patients with respiratory symptoms attending hospitals in the Lisbon area, between 2010 and 2018. Their bronchoalveolar lavage (BAL) specimens were submitted to the Group of Opportunistic Protozoa/HIV and Other Protozoa of Instituto de Higiene e Medicina Tropical (Lisboa, Portugal) with the purpose of routine diagnosis of PcP with the patients’ informed consent and according to the routine institutional procedures. The diagnosis of PcP was confirmed in 48 patients by visualization of the organism in BAL specimens using indirect immunofluorescence with monoclonal antibodies (IF/MAb) (MONOFLUO *Pneumocystis jirovecii*, BioRad^®^) and/or by *P. jirovecii*’s DNA detection through nested-PCR (nPCR) from the locus *mtLSUrRNA*, after DNA extraction (QIAamp, QIAGEN^®^). Twenty-eight patients whose specimens were negative for *P. jirovecii* were considered not infected (without PcP). All patients’ demographic data were kept confidential and coded for the research team.

All selected patient’s sera samples were analyzed through indirect ELISA for detection of circulation anti-*P. jirovecii* antibodies. Then, the optimization of AuNP-RSA conjugates interaction with anti-*P. jirovecii* antibodies was performed creating a pool of positive sera (positive sample) and a pool of negative sera (negative sample) using five serum specimens from patients with and without *P. jirovecii* infection, respectively.

The Instituto de Higiene e Medicina Tropical (Lisboa, Portugal) ethics committee approved the study’s protocol and waived informed consent as a retrospective observational study.

### ELISA for Detection of Anti-*P. jirovecii*’s Antibodies

The purified RSA were applied individually as antigenic tools in indirect ELISA to detect IgG and IgM anti-*P. jirovecii* in patient’s sera. Odd (test) column wells of the microtiter plate were coated overnight at 4°C with 50 μL of the RSA diluted to 5 μg.mL^–1^ in 0.05 M carbonate buffer (pH = 8.4). Even (blank) columns wells were coated in the same conditions with PBS 1x. After coating, the wells were washed once with PBS and blocked with 70 μL of 1% polyvinyl alcohol (PVA) for 1 h at room temperature (20–25°C). After blocking, PVA was removed from the plate without washing. Duplicates of each serum sample were analyzed in a test and blank well, under conditions (dilution, incubation time and temperature) determined by the Ig class to be detected and the RSA used as antigenic tool, being the specific protocols presented at Table 1. Finally, the optical densities (OD) were measured at 405nm and the mean OD of the blank wells was deducted to the mean OD of the test wells to obtain the final OD value for each sample.

**TABLE 1:** ELISA protocols and conditions for detection of IgG and IgM anti-*P. jirovecii* antibodies reactive against the RSA produced.

**Protocol**	**RSA applied as antigenic tool**			
	**Kex1**		**Msg**	
Application of 50 μL of serum diluted in PBS with 0.05% tween-20 and 5% BSA	1/20	1/40	1/60	1/140
Incubation	1 h at 37°C			
Washing	4x with PBS with 0.05% tween-20			
	1x with distilled water			
Application of 50 μL of anti-human immunoglobulin M (A2189, sigma^§^) diluted in PBS with 0.05% tween-20	1/1000		1/4000	
Application of 50 μL of anti-human immunoglobulin G (A2064, sigma^§^) diluted in PBS with 0.05% tween-20		1/3000		1/4000
Incubation	1 h at 37°C			
Washing	4x with PBS with 0.05% tween-20			
	1x with distilled water			
Application of 50 μL of substrate	4-nitrophenylphosphate sodium salt (1 mg.mL^–1^)			
Incubation	Overnight at 4°C	Overnight at 4°C	Overnight at 4°C	2 h at 37°C

Mann-Whitney-U non-parametric tests were used to examine the differences between the distribution of antibody titers in different patient categories with a significance level of 0.05, using the Statistical Package for Social Sciences (SPSS) version 20.0.

### Synthesis and Functionalization of Spherical Gold Nanoparticles

For synthesis and functionalization of gold nanospheres, all glassware was washed with aqua regia and rinsed thoroughly with deionized water followed by ultrapure water (18.2 MΩ⋅cm^–1^) before use.

Citrate capped spherical gold nanoparticles (AuNPs) were synthesized following a method previously described (Bastús et al., 2011). Briefly, 150 mL of a 2.2 mM citrate solution (1.06448, Merck^®^) was heated using an oil bath, under stirring. After the reflux was stablished, 1 mL of a 25 mM gold (III) chloride solution (484385, Sigma^®^) was added to the reaction vessel and let to react for 10 min. After these steps, a seeds suspension was obtained. Then, the resultant suspension was cooled down to 90°C, keeping the condenser fitted and the stirring conditions. An extra 1 mL of the same gold (III) solution was added and let to react for 30 min. After this period, this last step was repeated.

Later, the citrate capping was replaced by functionalization of the AuNPs with 11-mercaptoundecanoic acid (11-MUA, 450561, Aldrich^®^). This step involved adding 10 mM 11-MUA ethanolic solution, to attain a 11-MUA:AuNP ratio of 30,000. After an overnight incubation, the AuNPs were washed (3000 *g* for 30 min) to remove free 11-MUA in solution. The highest volume possible of supernatant was discarded and the pellet was resuspended in ultrapure water up to ∼5% of the initial volume. The final suspension was stored in the dark until use.

AuNPs were characterized by ultraviolet-visible spectroscopy (UV-Vis) before and after functionalization and by dynamic light scattering (DLS), electrophoretic light scattering (ELS) and nanoparticle tracking analysis (NTA) after the functionalization process. UV-Vis was performed in a Varian Cary 50 Bio spectrophotometer, using a quartz cell, with the suspension at an appropriate dilution. DLS and ELS measurements were performed three times for the same sample at 25°C, with light detection at 273° (DLS) and at 17° using the backscatter mode (ELS) of the Malvern Zetasizer NanoZS equipment. NTA was performed in a Malvern Nanosight NS300 (with a 642 nm laser module), with the analysis of 5 videos of 1 min each, captured in 5 different portions of the sample (still mode). These measurements were then merged in a single size distribution.

### Conjugation of AuNPs With the RSA

The antigen concentration to use in the conjugation process to guarantee the maximum coverage of the AuNPs surface but also the colloidal stability of the AuNP-RSA conjugates was optimized. For that purpose, AuNP-Msg and AuNP-Kex1 conjugates were formed through electrostatic interactions established between increased molar ratios of each RSA and the AuNPs in solution, as described previously (Guirgis et al., 2012; Cavadas et al., 2016; Almeida et al., 2018). A solution of 0.06nM AuNPs was incubated overnight (≈15 h) at 4°C with molar ratios ranging from 0 to 5000 of Msg and Kex1 stock solutions of 0.16 and 0.11 mg.mL^–1^, respectively. After conjugation, non-bound RSA were removed by centrifugation (5800 g for 5 min), separating the pellets containing the AuNP-RSA from the supernatant, to perform agarose gel electrophoresis (AGE). Agarose gels (0.3%) were prepared by heating agarose in TAE buffer (0.125 × pH = 8.4), and allowing the gel to form at room temperature. The AuNP-RSA conjugates pelleted after centrifugation were re-suspended in 13.5 μL of phosphate buffer (5 mM Na_2_HPO_4_, pH = 7.4) and 1.5 μL of glycerol (99%, Nzytech^®^) prior to loading. The gels were run at constant voltage of 180 V with a 21 cm electrode spacing for 20 min in TAE 0.125 × using the E865 CONSORT power supply. Digital pictures were acquired (WAS-LX1A Huawei P10 Lite camera) and processed through eReuss software (a gel analysis application freely available at https://github.com/lkrippahl/eReuss), providing an accurate measurement of the red bands migration in agarose, which allows the calculation of their electrophoretic mobility (Ferard, 1994; Almeida et al., 2018). Electrophoretic mobility [μ (μm.cm/V.s)] is defined as the observed rate of migration of a component [ν (μm/s)] divided by the electric field strength [*E* (V/cm)] in a given medium. In the case of AGE, a solid support medium, only apparent values can be determined (Ferard, 1994). Thus, the molar ratio in which the AuNP-RSA electrophoretic mobility reaches a plateau, corresponding to saturation of the AuNP surface with each RSA, was selected through duplicate experiments.

### Analysis of Human Sera Interaction With AuNP-RSA by Agarose Gel Electrophoresis

To avoid unspecific antibody binding to the AuNP-Msg and AuNP-Kex1 conjugates, bovine serum albumin (BSA) (AppliChem^®^) and Casein (Sigma^®^) were studied as blocking agents. A BSA and Casein stock solution at 1 mg.mL^–1^ were added to 0.06 nM AuNP-RSA conjugates in solution at increased molar ratios ranging from 0 to 10 with AuNP-Kex1 conjugates and from 0 to 50 with AuNP-Msg conjugates, producing AuNP-RSA-BSA and AuNP-RSA-Casein conjugates. The incubation was performed during 90 min at 4°C, the non-bound blocking agents were removed by centrifugation (5800 g for 5 min) and the pellets prepared for agarose gel electrophoresis. Similarly to what was done with the AuNP-RSA conjugates, the molar ratio plateau was selected through duplicate experiments for each blocking agent.

An optimal molar ratio of human serum was established in the same way, incubating 0.06 nM solutions of AuNP-RSA-Casein and AuNP-RSA-BSA conjugates with molar ratios ranging from 0 to 7.5 of the positive sera pool (80 mg.mL^–1^). The molar ratio in which the optimal coverage of the conjugate was obtained, was selected.

These serum molar ratios were applied in an AGE assay, where 0.06 nM AuNP-RSA solutions and 0.06nM AuNP-RSA-BSA and AuNP-RSA-Casein conjugates solutions were incubated for 90 min at 4°C with and without the human positive and negative sera pools. After incubation, the non-bound serum was discarded after centrifugation (5800g for 5 min) and the electrophoretic mobility of each conjugate was established. This assay was performed in triplicate and the analysis of the differences between the electrophoretic mobility of each conjugate formed was evaluated and used to established the optimal conjugate (AuNP-RSA or AuNP-RSA-BSA or AuNP-RSA-Casein) to be used in the LFIA development.

### Assembly of LFIA Strips for Detection of IgM Anti-*P. jirovecii* Antibodies in Human Sera

For LFIA development, a starter kit from Advanced Microdevices, Ambala Cantt, India, was used. This kit offers: two types of cellulose fiber absorbent pads with different thickness (AP-045 with 0.4 mm and AP-080 with 0.8 mm); three types of nitrocellulose membranes (NM) fixed on a plastic backing, with different protein binding capacities (type CNPF, a low protein binding membrane with pore sizes of 8 and 10 μm; type CNPC, a high protein binding membrane with pore sizes of 12 and 15 μm; and type CNPH, the highest protein binding membrane with wicking times of 70, 90, 150, and 200 s); two glass fiber sample pads, one without any specific pre-treatment (GFB-R4) and a second one pre-treated with buffers and detergents to help prevent non-specific binding of sample components to the pad (GFB-R7L); and two conjugate pads, a polyester matrix without any specific pre-treatment (PT-R5) and another one pre-treated with buffers for uniform movement of gold nanoparticle conjugates (PT-R7).

#### Absorbent Pad and Membrane Selection

To perform this selection, dipsticks composed by the nitrocellulose membrane (NM) and the absorbent pad were tested. Membranes were manually cut into 6 × 0.5 cm sections. The control and test lines were spotted manually, in a circle or in a line, by depositing 0.03 mg.mL^–1^ of anti-RSA antibodies (anti-Msg or anti-Kex1, depending on the RSA present in the conjugate) and 0.001 mg.mL^–1^ of anti-human IgM antibodies (I-0759, Sigma^®^), respectively. Different dilutions in Tris buffer (10 mM Tris-HCl, pH = 7) for the control antibodies, ranging from 0 to 1/4, and for the test antibodies, ranging from 0 to 1/10, were studied. The membranes dried for 30 min at room temperature before use. A blocking process of the membranes was also tested with an additional step of 30 min incubation at room temperature with skin milk (2%, Sigma^®^), followed by three washes with PBS with 0.05% Tween-20 and dried at 37°C during 30 min. At the top of the membrane, a 3 × 0.5 cm section of absorbent pad (AP-045 or AP-080) was attached with 1–2 mm overlapping with the membrane. A solution of 0.72 nM AuNP-RSA-Caseín conjugates was incubated with appropriate molar ratio of positive serum for 90 min at 4°C and then, the dipsticks composed by the NM and the absorbent pad were immersed into 50 μL of this solution in a tube, with the absorbent pad side up for 2 min. Driven by capillary forces, the liquid migrated up the membrane into the absorbent pad. The selection of the optimal set of membrane/absorbent pad was based on visually inspection of test and control line results.

#### Conjugate Pad, Sample Pad and Sample Buffer and Dilution Selection

To perform this selection, full LFIA strips were assembled and tested. Conjugate release pads available were manually cut into 1 × 0.5 cm sections and tested with and without pre-treatment with PBS containing 5% sucrose, 1% BSA and 0.5% Tween 20. Then, they were saturated with 15 μL of conjugate concentrations ranging from 0.2 to 4.6 nM. These conjugate pads dried for 2 h at 37°C before assembling to the membrane/absorbent pad set (see above) and then attached to the bottom of the strip (at the origin of the sample flow), with 1–2 mm overlapping with the membrane. The sample pads were also manually cut into 3 × 0.5 cm sections and tested with and without prior saturation with 0.03% anti-human immunoglobulin G (2040-04, Sigma^®^) and dried overnight at room temperature, before assembling at the bottom of the strip, overlapping almost completely with the conjugate pad. After the whole LFIA strip was assembled, 200 μL of positive serum diluted from 0 to 1/100 into phosphate buffer (5 mM Na_2_HPO_4_, pH = 7.4), phosphate buffer with 0.05% BSA and 0.05% Tween-20, phosphate buffer with 1% BSA and 1% Tween-20 or PBS with 0.05% Triton-X-100, was added to the sample pad. Driven by capillary force, the sample migrated up the sample pad to the conjugate pad and to the membrane into the absorbent pad. After 10 min, the selection of the optimal set of conjugate pad/sample pad and optimal sample buffer/dilution conditions was based on a visually inspection of test and control line results, on the manufacturer’s recommendation for the uniform movement of gold nanoparticles conjugates and on quantification of color intensity in test and control lines.

#### Quantification of Color Intensity

The quantification of color intensity in test and control lines was made in strips tested before and after pre-treatment of conjugate/sample pads, in strips tested with different positive sample dilutions and in strips tested with the final optimal conditions. Digital pictures of the optimized strips were acquired (WAS-LX1A Huawei P10 Lite camera) and eReuss (a gel analysis application freely available at https://github.com/lkrippahl/eReuss) parameters were adapted for color intensity quantification. To set the image processing, in the band color droplist of the software, the average color of the test and control lines was selected, forcing the software to ignore the color channel in which the lines have a light intensity closer to the background. This step ensures that the intensity value given by the software for each line results from the previous elimination of the background. Then, in the image clipping set, the NM region with both test and control lines results was selected for processing. The software, for each defined vertical strip, sums the color intensity of the pixels in horizontal sections giving a plot with the heights of each point per line. In band profiling step, the conditions in which we intend to measure the peaks of color intensity in the different control/test lines were set. Two Gaussians were fitted per strip, one for each test or control line, with a minimum height value of 5% of the image brightness range, to set the cut-off value of intensity. The remaining parameters were left with the default settings. The software identified the peaks by iteratively fitting a Gaussian distribution to the maximum intensity value in the curve, subtracting that distribution and repeating until either the number of Gaussians was reached or the maximum value falls below the minimum height parameter. A final report with the summarized results was obtained and analyzed for selection of the optimal sample dilution and the need for sample/conjugate pad pre-treatments.

### LFIA Strips Testing With Clinical Samples

The viability of the prepared immunochromatographic strips as tools for detection of anti-*P. jirovecii* antibodies was tested, in triplicate experiments, by loading human serum pools from patients with and without PcP.

In the case of strips in which the conjugates were composed by AuNP-Msg-Casein, 200 μL of positive/negative human serum pools, diluted 1:50 in phosphate buffer with 0.05% BSA and 0.05% Tween-20, were added to the sample pad. In strips with adsorbed AuNP-Kex1-Casein conjugates, 200 μL of positive/negative human serum pools, diluted 1:20 in phosphate buffer with 0.05% BSA and 0.05% Tween-20, were used instead. Driven by capillary forces, the samples migrated up the conjugate pad to the membrane into the absorbent pad and, during 10 min, the test results were evaluated visually. Further quantification of color intensity in test and control lines of each strip was performed using the eReuss software.

## Results

### Design, Expression, and Purification of Msg and Kex1 RSA

The Msg RSA was designed as previously described (Tomás et al., 2016).

The Kex1 RSA consists of 110 amino acids composed by three regions with high-predicted antigenicity and reactivity from *P. jirovecii*’s Kex1 entire sequence (see Supplementary Figures S1–S6), that were chosen and synthesized interconnected by two “linkers” of five glycine residues and expressed with a tail of six residues of histidine. In this RSA, epitope 1 is coded by Kex1_104__–__134_ amino acids, epitope 2 by Kex1_467__–__501_ amino acids and epitope 3 by Kex1_725__–__758_ amino acids, according to GenBank accession numbers AAN12365.1 and AAM97495.1 (see Supplementary Figure S7; Kutty and Kovacs, 2003).

**FIGURE 2:**
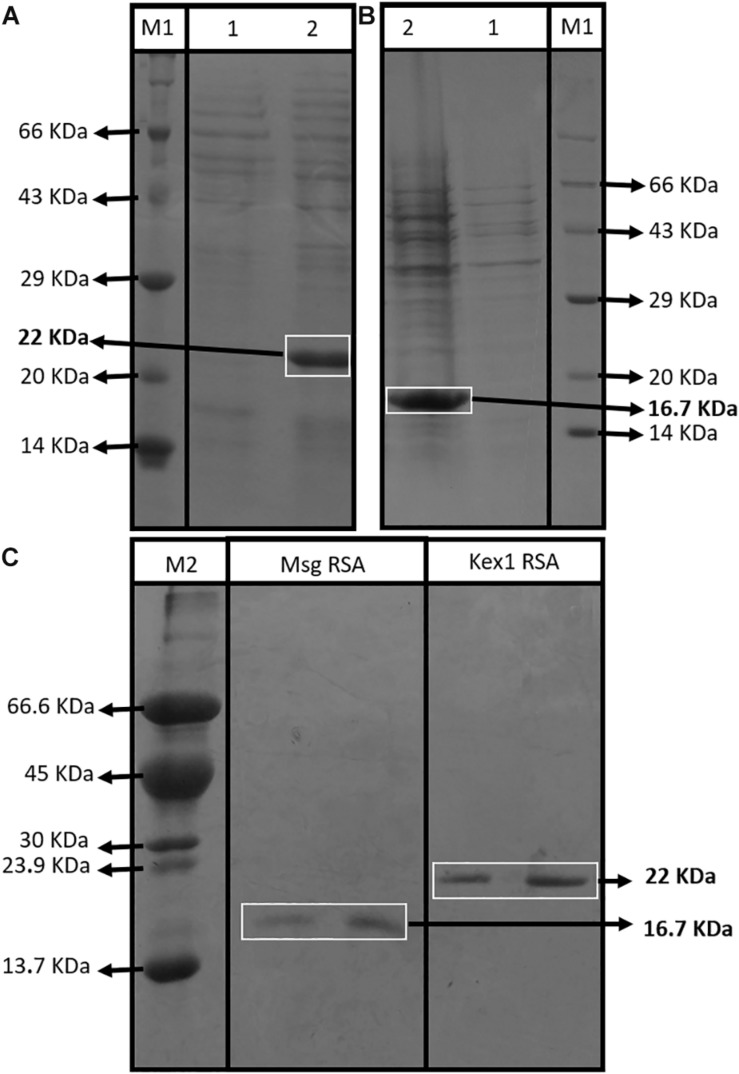
Assessment of the RSA expression and purification process. SDS-PAGE profiles of non-induced (1) and induced (2) *E. coli XJb (DE3)* cells transformed with the expression vector with the Kex1 RSA **(A)** and the Msg RSA **(B)**; and SDS-PAGE profiles of purification products of the Msg RSA and Kex1 RSA, after IMAC protocol **(C)**. Two different protein molecular weight standards were used, the Roti^§^ -Mark standard from Carl-Roth^§^ (M1) and one produced “in house” (M2). The bands of interest and their theoretical molecular weight are highlighted (16.7 kDa for Msg RSA and 22 kDa for Kex1 RSA).

The SDS-PAGE analysis showed that both RSA were expressed with their predicted molecular sizes (16.7 kDa for Msg RSA and 22 kDa for Kex1 RSA) after IPTG induction (Figures 2A,B) and that they were successfully purified by immobilized metal-ion affinity chromatography (IMAC) (Figure 2C). Indirect ELISA using anti-polyhistidine antibodies were performed to optimize the purification process, ensuring that the RSA were detected during the elution phase and not during column washing after sample application (see Supplementary Figure S8).

### Detection of Serum Anti-*P. jirovecii* Antibodies

**FIGURE 3:**
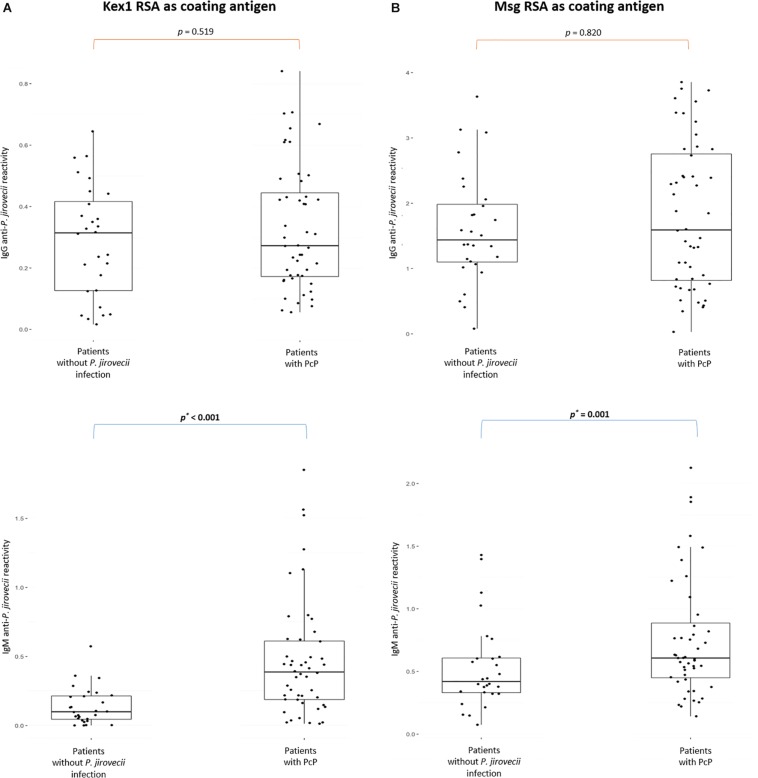
Boxplots with IgG and IgM anti-*P. jirovecii* levels results across patients with PcP and patients without *P. jirovecii* infection. Simple boxplots showing the reactivity levels (OD at 405 nm) of IgG and IgM anti-*P. jirovecii* antibodies detected by ELISA protocols applied with the Kex1 RSA **(A)** and Msg RSA **(B)** as coating antigens, in all sera specimens of patients analyzed in the study. The statistic values (*p*) representing the statistical significant difference from Mann Whitney-U tests performed between patient’s groups are presented.

Two different IgG and IgM ELISA were developed, according to the protocols presented in Table 1, using Kex1 RSA and Msg RSA as coating antigens. The results of the distribution of the IgG and IgM anti-*P. jirovecii* levels across patients with PcP and without *P. jirovecii* infection are represented in Figure 3.

Both RSA showed applicability in the detection of specific IgG and IgM anti-*P. jirovecii* antibodies. However, IgG ELISA showed inability to distinguish patients with PcP from patients without *P. jirovecii* infection as the median levels of the Igs detected in these patient’s groups were not statistically different (*p* > 0.05). Yet, IgM ELISA with both Kex1 RSA and Msg RSA demonstrated ability to distinguish patients with the disease from patients without *P. jirovecii* infection. With the Kex1 RSA, the median levels of IgM anti-*P. jirovecii* detected by ELISA was 0.3871 in patients with PPc and 0.0997 in patients without *P. jirovecii* infection, which were considered statistically different (*p* < 0.002). With the Msg RSA, the differences between the median levels of IgM anti-*P. jirovecii* detected by ELISA in patients with and without the disease were also considered statistically significant (*p* = 0.001), and the values were 0.6076 in patients with PPc and 0.4195 in patients without *P. jirovecii* infection.

### Characterization of Gold Nanoparticles

Gold nanoparticles were characterized by UV-Vis, DLS, ELS, and NTA.

The UV-Vis spectrum of the citrate-capped AuNPs (as synthesized) shows a localized surface plasmon resonance (LSPR) band with its maximum at 526 nm. From the UV-Vis spectrum data (Abs_LSPR_ and Abs_450_), it was determined that the batch of AuNPs had a concentration of 0.2 nM with an average size of 39 nm (Haiss et al., 2007). After functionalization with 11-MUA (see Supplementary Figure S9A) the hydrodynamic size data obtained from DLS showed a Z-Average of 46.2 ± 0.2 nm. The zeta-potential value obtained by ELS was -36 ± 1 mV, indicating a high colloidal stability. The hydrodynamic diameter distribution obtained by NTA (see Supplementary Figure S9B), presented an average of 51.0 ± 3.8 nm and a mode of 41.7 ± 2.9 nm. The mode is down shifted by 9.3 nm compared to the average, since the aggregates (especially noticeable in the distribution between 60 and 90 nm) contribute for the mean value.

### Gold Nanoparticle-*P. jirovecii*’s RSA Conjugates (AuNP-RSA)

**FIGURE 4:**
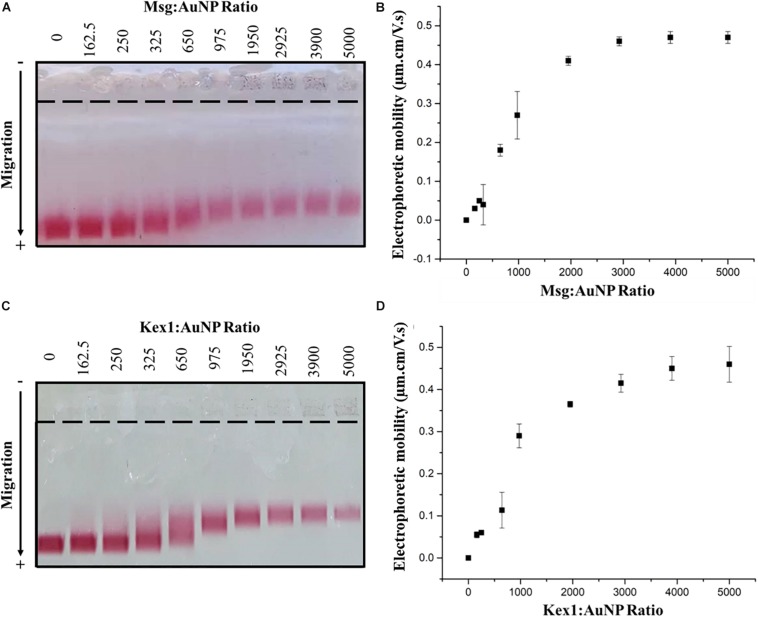
AuNP-RSA conjugates formation. Agarose gel electrophoresis at increasing ratios of Msg RSA to AuNPs **(A)** and Kex1 RSA to AuNPs **(C)**, showing the migration of each conjugate bands toward the positive electrode. Differences between the electrophoretic mobility of the AuNPs alone and AuNP-Msg **(B)** or AuNP-Kex1 **(D)** conjugates at increasing ratios, calculated from the migration distances in gel. The nine ratios (162.5–5000) correspond to protein concentrations of 9.75, 15, 19.5, 39, 58.5, 117, 175.5, 234, and 300 nM. Standard deviation bars correspond to duplicate experiments.

The functionalized AuNPs produced were conjugated to Msg (AuNP-Msg) or Kex1 (AuNP-Kex1) RSA of *P. jirovecii*, in order to synthesize probes for the detection of IgM anti-*P. jirovecii* antibodies in sera of patients with PcP. Agarose gel electrophoresis assays were used to characterize the AuNPs before and after conjugation with the RSA (Guirgis et al., 2012; Cavadas et al., 2016; Kim et al., 2016; Almeida et al., 2018). As shown in Figures 4A,C, the migration distance of the RSA-conjugated particles (AuNP-Msg and AuNP-Kex1), compared to the non-conjugated AuNPs, decreases as the RSA:AuNP ratio increases. However, when the amount of RSA reached 175.5 nM (ratio 2925:1), the addition of more RSA no longer decreased significantly the mobility of the conjugates and a plateau began to form. Therefore, a RSA:AuNP ratio of 2925:1 was stablished as the optimum to produce AuNP-RSA conjugates in which RSA molecules fully cover the AuNPs. For these RSA:AuNP ratios of 2925:1, the difference between the calculated electrophoretic mobility (Δμ) of AuNP alone and AuNP-RSA conjugates was 0.46 ± 0.01 μm.cm/V.s for AuNP-Msg conjugates and 0.42 ± 0.02 μm.cm/V.s for AuNP-Kex1 conjugates (Figures 4B,D).

### Blocking of AuNP-RSA Conjugates for Reaction With Human Sera

In order to avoid unspecific interactions with human sera, increasing the specificity of the interaction between the conjugates and the target antibodies, blocking of the AuNP-RSA conjugates was assessed with BSA or with casein, two well know proteins used routinely for this purpose in immunoassays (Binder and Isler, 2013). The electrophoretic mobility of AuNP-Msg conjugates incubated with increasing ratios of BSA or casein was determined by AGE (see Supplementary Figure S10). A plateau was observed for a BSA:AuNP-Msg ratio of 20, so that value was chosen for future experiments. In the case of AuNP-Kex1 conjugates, since there is no observable interaction of the blocking agents with these conjugates, no particular BSA:AuNP-Kex1 and Casein:AuNP-Kex1 ratios were found (see Supplementary Figure S11). However, to evaluate the interaction of human sera with conjugates with and without blocking agent, a ratio of 2:1 of casein to AuNP-Kex1 was used.

The blocked and unblocked AuNP-RSA conjugates were further assessed by AGE to characterize their ability to interact with human sera from patients with and without *P. jirovecii* infection. Based on the same type of AGE experiments, in which several molar ratios of the positive human serum pool to blocked AuNP-RSA conjugates were evaluated, a human serum ratio of 4.55 was selected for further experiments (see Supplementary Figures S12, S13).

AuNP-RSA, AuNP-RSA-BSA, and AuNP-RSA-Casein conjugates were incubated with and without positive (PosSerum) and negative (NegSerum) serum pools at the selected ratio and then assessed by AGE (see Supplementary Figure S14). Results suggest that there were still free spaces on the surface of the AuNP-Msg conjugates that were blocked by casein and by BSA, causing a decrease in the electrophoretic mobility of the conjugates in the absence of serum (see Supplementary Figures S14A,B). However, in the case of AuNP-Kex1 conjugates (see Supplementary Figures S14C,D), the electrophoretic mobility of the conjugates before serum interaction, is very similar in the presence or absence of any blocking agent, which reinforces the idea that the blocking step is not as crucial for these AuNP-Kex1 conjugates as it is for the AuNP-Msg conjugates, as previously observed (see Supplementary Figures S10, S11). Nevertheless, the blocking step was maintained for both AuNP-RSA conjugates, in order to avoid the event of non-specific interactions with human sera components. For this purpose, casein appeared to be more effective, as non-specific interactions between the negative serum and the conjugates were lower in the presence of this blocking agent than in the presence of BSA. That can be verified by a decrease in the migration shift between the blocked conjugates, before and after interaction with the negative serum (see Supplementary Figure S14: a shift of 0.13 μm.cm/V.s in AuNP-Msg-Casein conjugates against a shift of 0.42 μm.cm/V.s in AuNP-Msg-BSA conjugates; a shif of 0.23 μm.cm/V.s in AuNP-Kex1-conjugates against a shift of 0.39 μm.cm/V.s in AuNP-Kex1-BSA conjugates).

### Interaction Between Human Sera and AuNP-RSA-Casein Conjugates

**FIGURE 5:**
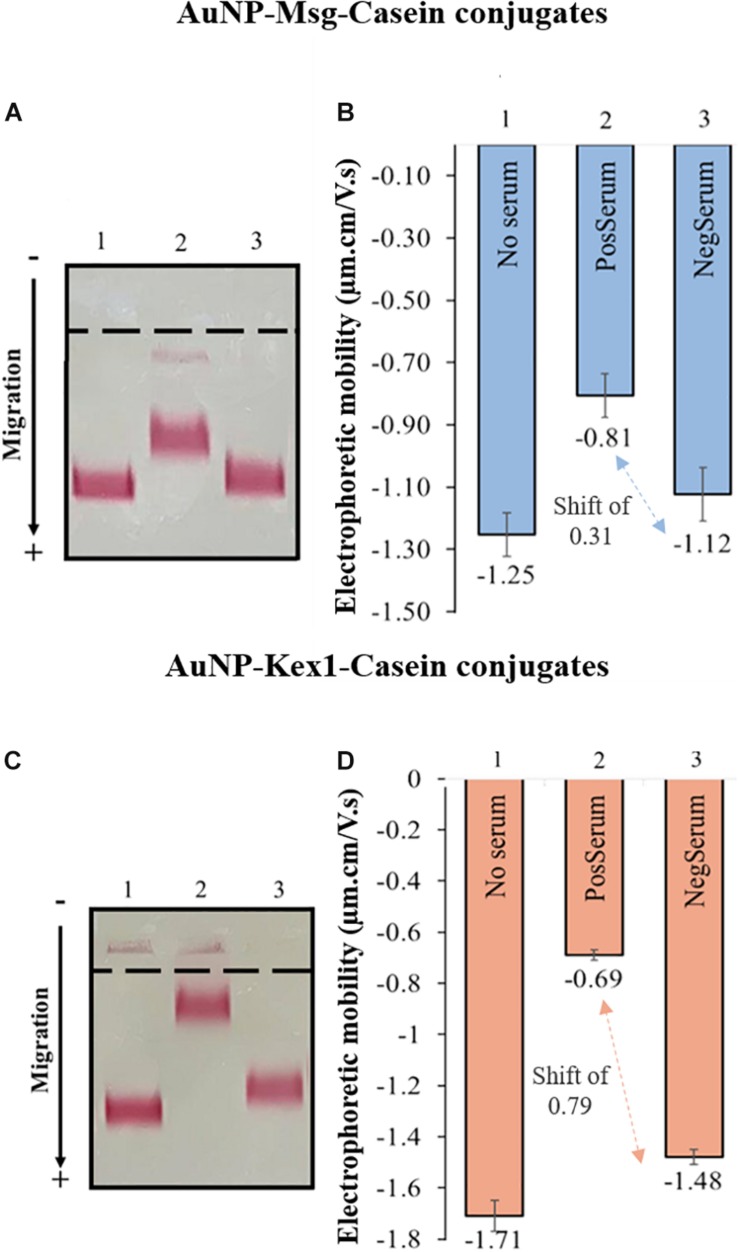
Interaction of AuNP-Msg-Casein **(A,B)** and AuNP-Kex1-Casein conjugates **(C,D)** with sera pools from patients with and without *P. jirovecii* infection. **(A,C)** Agarose gel electrophoresis of AuNP-RSA-Casein conjugates before interaction with serum (1) and after interaction with positive (2) and negative (3) serum samples. **(B)** Electrophoretic mobility of the AuNP-RSA-Casein conjugates before interaction with human sera (No Serum) and after interaction with the positive human sera (PosSerum) and negative human sera (NegSerum). Shifts (μm.cm/V.s) between blocked conjugates with positive and with negative sera are indicated, evidencing the binding of antibodies present in the positive serum. Standard deviation bars correspond to triplicate experiments.

The interaction between AuNP-RSA-Casein conjugates and human sera from patients with and without *P. jirovecii* infection was assessed by AGE and is represented in Figure 5. Results shows a consistent detection of a migration shift between AuNP-RSA-Casein conjugates that interacted with sera from PcP patients and conjugates that interacted with sera from patients without *P. jirovecii* infection (0.31 μm.cm/V.s in the case of AuNP-Msg-casein conjugates and 0.79 μm.cm/V.s in the case of AuNP-Kex1-casein conjugates). The presence of these shifts in AGE assays are a proof-of-concept for the LFIA to be developed: in fact, anti-*P. jirovecii* antibodies present in PcP patient’s sera specifically interact with both RSA, binding to the AuNP-RSA-Casein conjugates and decreasing their migration distance in the gel. Additionally, no significant interactions between AuNP-RSA-Casein conjugates and non-infected patient’s sera occur, since the migration distance of these conjugates before and after contact with the negative sample is similar, considering all the experiments performed.

Results also show that the distance migration shift between conjugates that interact with the positive sera and conjugates that interact with negative sera is bigger in the presence of AuNP-Kex1 conjugates (up to 2.5x), suggesting that this RSA is a better antigenic tool for anti-*P. jirovecii* antibodies detection than the Msg RSA.

### Assembly and Optimization of LFIA Strips Using AuNP-RSA-Casein Conjugates

For LFIA strips development, a commercial starter kit was used (see section Materials and Methods for a detailed description of the components). LFIA’s components selection was based on manufacturer’s advices and visual inspection of test and control lines results (data not shown).

#### Absorbent Pad and Membrane Selection and Optimization

By visual inspection, it was observed that all NM can be used successfully without a previous blocking step. Additionally, the signal in the control and test lines appeared to increased proportionally with pore diameter and the wicking time of the NM. However, as membranes type CNPH are presented by the manufacturer as the NM with the highest protein binding capacity, the one with the longest wicking time (200 s) was the one chosen for the LFIA development.

No visual differences were noted in CNPH200 NM dipsticks results using one or the other absorbent pad available in the kit. However, as absorbent pad 045 is thinner, it allows a slower migration along the NM, which may improve the number of interactions in the control and test lines. Therefore, it was the absorbent pad selected for LFIA development.

The selection of the control and test antibodies dilutions was made in order to obtain uniform signals in both lines. By visual inspection (data not shown), a dilution of 1/2 in Tris buffer for the control antibodies and no dilution for the test antibodies were selected.

#### Conjugate and Sample Pad Selection and Optimization

In the case of conjugate pads available, as the manufacturer states that the PT-R7 pad was pre-treated for uniform movement of gold nanoparticles conjugates, unlike the PT-R5 pad, the PT-R7 pad was the one selected. Concerning the AuNP-RSA-Casein conjugates concentration to be used, a colloidal solution of 2.4 nM was stablished as sufficient to provide a visual interpretation of the test results (data not shown).

Regarding the sample pad selection, as GFB-R7L pads were pre-treated by the manufacturer with detergents and buffers that decreased sera non-specific binding to the pad, they allowed better visual interpretation of the results than the GFB-R4 untreated pad, and that’s why GFB-R7L pad was the one chosen.

**FIGURE 6:**
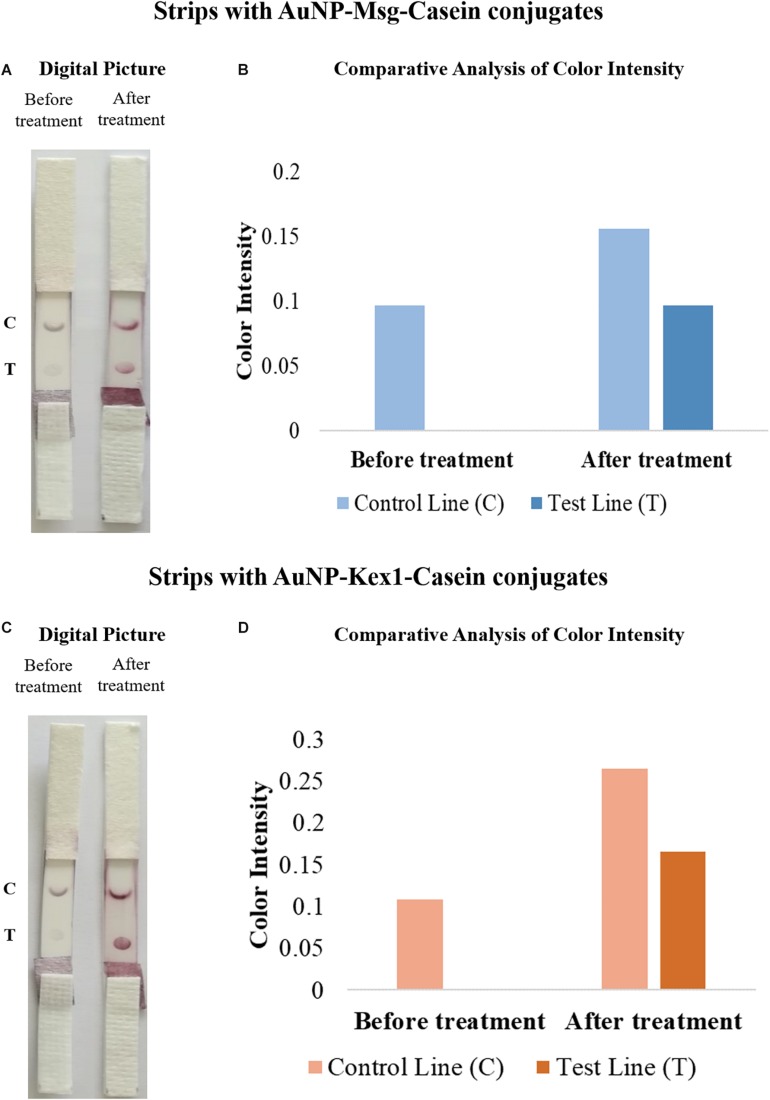
Comparative analysis of the results from LFIA strips containing AuNP-Msg-Casein conjugates **(A,B)** and AuNP-Kex1-Casein conjugates **(C,D)** in the presence (after treatment) and absence (before treatment) of conjugate and sample pad pre-treatments. **(A,C)** Digital pictures of strips before and after conjugate pad treatment with a buffer (5% sucrose, 1% BSA and 0.5% Tween 20) and sample pad treatment with 0.03% anti-human immunoglobulin G. From bottom to top, strips were composed by the sample pad, the conjugate pad, the nitrocellulose membrane with the test (T) and control (C) lines and the absorbent pad. **(B,D)** Quantification of color intensity of the control and test lines, where the intensity shown in each line corresponds to the maximum height of the Gaussian line fitted by eReuss software.

To improve signal intensity on both test and control lines, pre-treatments of the selected conjugate pad (with a buffer containing sucrose, BSA and Tween 20) and sample pad (with anti-human immunoglobulin G) were performed. The color intensity of test/control lines in strips before and after treatment was assessed visually and quantified by the eReuss software, and the results are presented in Figure 6. Results show that pre-treatments steps enhanced visual signal of both test and control lines (Figures 6A,C), which was confirmed by color quantification, showing higher peaks of color intensity in both lines after these treatments (Figures 6B,D).

#### Sample Buffer and Dilution Selection

**FIGURE 7:**
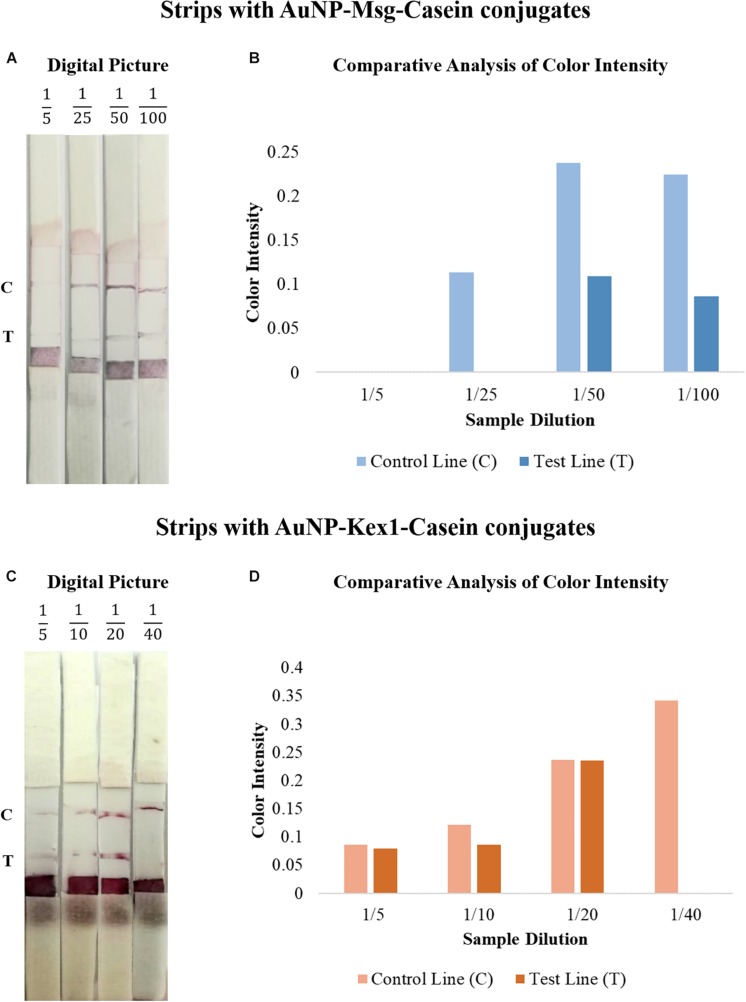
Comparative analysis of the results from LFIA strips containing AuNP-Msg-Casein conjugates **(A,B)** and AuNP-Kex1-Casein conjugates **(C,D)**, after applying the positive sample in different dilutions. **(A,C)** Digital pictures of LFIA strip results with sample dilutions ranging from 1:5 to 1:100 in Msg LFIA strips and ranging from 1:5 to 1:40 in Kex1 strips. From bottom to top, strips were composed by the sample pad, the conjugate pad, the nitrocellulose membrane with the test (T) and control (C) lines and the absorbent pad. **(B)** Quantification of color intensity of the control and test lines, where the intensity shown in each line corresponds to the maximum height of the Gaussian line fitted by eReuss software.

By visual inspection of strip results (data not shown), phosphate buffer with 0.05% BSA and 0.05% Tween-20 was selected as the optimal sample buffer. Then, final dilutions of 1:50 and 1:20 of sera samples were established as optimal for LFIA with AuNP-Msg-Casein conjugates and AuNP-Kex1-Casein conjugates, respectively, through visual inspection and color intensity quantification of strip test results, represented in Figure 7. Although higher color intensities were expected at lower sample dilutions, these results were reproducible and may be associated with an agglutination phenomenon, further addressed in the Discussion section.

### Optimized LFIA Strips Testing With Human Sera Pools

**FIGURE 8:**
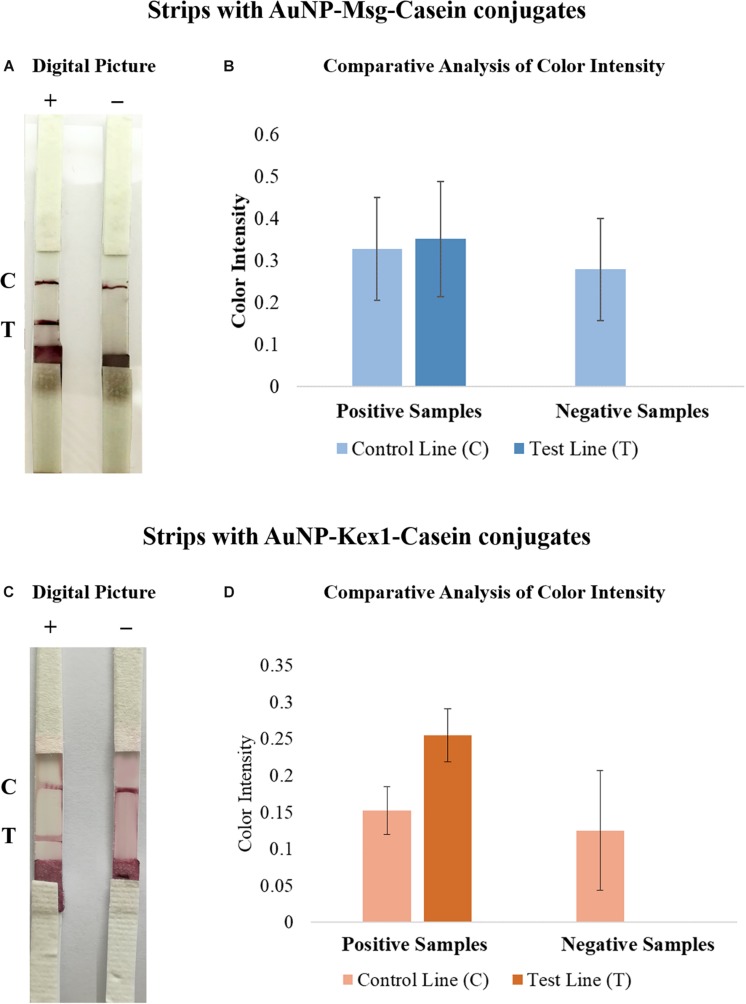
Comparative analysis of the results from LFIA strips containing AuNP-Msg-Casein conjugates **(A,B)** and AuNP-Kex1-Casein conjugates **(C,D)**, after elution of a positive (+) or a negative (-) sample. **(A,C)** Digital pictures of LFIA strip results. From bottom to top, strips were composed by the sample pad, the conjugate pad, the nitrocellulose membrane with the test (T) and control (C) lines and the absorbent pad. **(B,D)** Quantification of color intensity of the control and test lines present in all replicates, where the intensity shown in each line corresponds to the maximum height of the Gaussian line fitted by eReuss software and the error bars represent the standard deviation values.

After optimization, LFIA strips were tested with sera pools from patients with (positive sample) and without (negative sample) PcP, in triplicate experiments (see Supplementary Figure S15). Visually, 3 min after sample addition it was already possible to detect the presence of a colored line in the control zone on strips with the negative sample and two colored lines, in the control and test zones, on strips with the positive sample. The results remained invariable 10 min after the end of the elution process, i.e., solvent reaching the absorbent pad (Figures 8A,C).

The software used for color intensity analysis was unable to detect color on the test lines of the strips with negative samples, and detect similar color intensity for the control and test lines on the strips with positive samples (Figures 8B,D).

## Discussion

Nowadays, there is a demand to find point-of-care diagnostic tests that enable fast and inexpensive screening/diagnosis of infectious diseases, to improve disease control and retrenchment of healthcare systems costs worldwide. In the case of *Pneumocystis* pneumonia, in which there are no specific clinical, radiologic or gasometric findings and none of the serologic biomarkers studied until now showed to be highly-specific of the disease (Morris and Masur, 2011; Esteves et al., 2015; Matos and Esteves, 2016; Tomás and Matos, 2018), this has been a challenge. Thus, current diagnosis techniques depend on the direct or indirect detection of the pathogen in respiratory specimens, which makes them dependent on costly and invasive procedures.

Recently, studies showed that a technology based on synthetic amino acid sequences, designed to hold more than one reactive region of the selected antigens, could enhance the immunological diagnosis of *Toxoplasma gondii* (Dai et al., 2012, 2013). Therefore, in our previous study, this research group designed a recombinant synthetic antigen (RSA) with three antigenic regions of the Msg protein, in order to standardize and enhance the detection of reactive antibodies against *P. jirovecii* (Tomás et al., 2016). In this single antigenic tool, several proven reactive and conserved fragments of the Msg were used, improving its immunogenic power and consequently its application as an anti-*P. jirovecii* antibody detection tool (Tomás et al., 2016). In the present study, this RSA was used in combination with a new RSA, produced based on the immunogenic behavior of *P. jirovecii* Kex1 protein. This second RSA was also designed to hold more than one reactive region of *P. jirovecii* Kex1 protein, in order to increase the sensitivity and specificity of the serological approach. The idea of using this new protein (Kex1), encoded by a single copy gene (Kutty and Kovacs, 2003), emanate from the recent reports that support its role in the protection of PcP (Gingo et al., 2011; Kling and Norris, 2016) and to counteract the genetic variation that the Msg protein may present during infection (Hauser, 2019).

Both RSA were obtained with high purity (Figure 2C), and were applied as antigenic tools in different ELISA assays (Table 1) to assess whether specific anti-*P. jirovecii* antibodies can be detected in human sera at the time of patient’s presentation with symptomatology compatible with PcP. Thus, 76 serum specimens collected at the time of patient’s BAL procedure for PcP routine diagnosis were analyzed by these optimized indirect ELISA with both RSA, for detection of IgG and IgM anti-*P. jirovecii* antibodies. IgG ELISA results showed that, even though IgG response is detected with both RSA, it is not possible to distinguish patients with PcP from patients without *P. jirovecii* infection by their IgG levels (Figure 3). That may be explained by previous PcP events or by the reports of *P. jirovecii* colonization in patients presenting diverse levels of immunodeficiency, primary respiratory disorders, or even in the immunocompetent general population (Wakefield et al., 2003; Medrano et al., 2005; Morris and Norris, 2012), which could lead to the production of memory cells that could induce IgG antibodies production throughout the individual’s life.

Yet, IgM ELISA using each RSA as a coating antigen (Figure 3), showed successful application in the serodiagnosis of PcP, as anti-*P. jirovecii* levels detected, unlike IgG levels, were significantly different between patients with and without the disease (*p* ≤ 0.001). These results corroborate what had previously been verified in studies with mice, where it was suggested that the IgM isotype has a predominant role in shaping the earliest steps in recognition and clearance of *P. jirovecii* infection, since IgM-deficient mice showed to be more susceptible to PcP progression (Rapaka et al., 2010). These results suggest that IgM anti-*P. jirovecii* antibodies seems to be a possible serological biomarker for active PcP diagnosis, which could provide a major improvement over the current diagnosis standards.

Taking this into consideration, the innovative character of the present study stems from the use of these RSA in association with gold nanoparticles with tunable bright colors, to design a point-of-care platform for PcP diagnosis based on a solid-phase (strip-based) test. The LFIA developed relies on the ability of AuNPs to interact with the RSA to form conjugates that are used as recognition tools capable of interacting with IgM anti-*P. jirovecii* antibodies present in the serum of patients with PcP (Figure 1). Although the traditional LFIA schemes for antibody detection uses AuNPs conjugated with an immunoglobulin-binding protein and the pathogen’s antigen immobilized in the analytical zone (test line), some studies have shown that a less conventional scheme using AuNPs directly conjugated with pathogen’s antigen molecules for detection of serum antibodies can achieve greater diagnostic sensitivity (Sotnikov et al., 2015, 2018). This increase in sensitivity is achieved by targeting the capture of the antibodies of interest in the conjugation process, due to the specific interaction of those antibodies with the corresponding antigens present in the conjugates. Thus, as in this study we sought to detect IgM, a class of immunoglobulins whose serum levels remain elevated for a short period of time during infection, less conventional AuNP-antigen conjugates were chosen for LFIA development. Additionally, ELISA results have shown that both Msg and Kex1 RSA are able to interact specifically with anti-*P. jirovecii* IgM antibodies in patient’s sera samples. Then, two LFIA strips for the detection of anti-*P. jirovecii* antibodies in human sera were developed, based on AuNP-Msg and AuNP-Kex1 conjugates.

Lateral flow technology is well suited to point-of-care diagnostics because it is robust and inexpensive, not requiring power, a cold chain for storage and transport, or specialized reagents (O’Farrel, 2013). This is possible because all necessary materials and reagents are prepared to be stable and ready to use at the time of sample application and the use of AuNPs ensures a visual interpretation of the results, without the need for any detection instrument.

Therefore, in this study, we synthetized MUA-capped AuNPs with a large diameter (≈40 nm, see Supplementary Figure S9) to obtain higher color intensity in the test/control lines with lower AuNPs concentrations (Haiss et al., 2007; Santra et al., 2017), which also helps to guarantee the low cost of the test. Additionally, the 11-MUA ligand is known to favor electrostatic protein conjugation with AuNPs (Gomes et al., 2012), which helped AuNPs conjugation with the *P. jirovecii*’s RSA and the formation of AuNP-Msg and AuNP-Kex1 conjugates.

These AuNP-RSA conjugates were characterized and optimized through AGE assays which allow the separation of the conjugates according to their differences in size and surface charge (Guirgis et al., 2012; Franco and Pereira, 2013; Cavadas et al., 2016; Kim et al., 2016; Almeida et al., 2018). Analysis of AuNP-RSA conjugates with increasing RSA:AuNP molar ratios after electrophoresis (Figures 4A,C) showed that as more RSA is adsorbed at the AuNPs surface, the formed conjugate migrates less in the agarose gel. This is consistent with increases in size, which decreases their electrophoretic mobility, and decreases in negative charge due to shielding induced by RSA coverage of the AuNPs, decreasing their ability to migrate toward the positive electrode. It was possible to define a plateau for the mobility of the AuNP-RSA conjugates (Figures 4B,D), considering the standard deviation values, that corresponds to saturation of the AuNPs surface with the RSA. So, these assays confirmed the formation of a persistent RSA corona around the AuNPs and allowed the selection of an optimal molar ratio of ca. 3000 RSA per AuNP for coverage of AuNPs with both RSA.

The ability of AuNP-RSA conjugates to interact with specific anti-*P. jirovecii* antibodies from human sera was also evaluated by AGE. The purpose of these assays was to establish a proof-of-concept for the LFIA test, demonstrating that these conjugates are indeed capable of functioning as anti-*P. jrovecii* antibody recognition tools. To achieve this goal, AuNP-RSA conjugates were incubated with human serum before and after treatment with BSA and casein (see Supplementary Figure S14), which are two non-antibody-reactive blocking agents that are usually applied as immunoassay blockers (Binder and Isler, 2013). This step was performed to ensure blockage of non-specific binding sites available on the surface of AuNPs after saturation with the RSA, so that non-specific interactions between AuNPs and serum proteins were minimized. For both AuNP-RSA conjugates, casein proved to be the most effective blocking agent, reducing non-specific interactions between human sera and those conjugates (see Supplementary Figure S14). However, even in the presence of a pre-blocking step with casein, there is some residual interaction between the negative serum and the conjugates (Figure 5). This event can be due to the presence of IgG anti-*P. jirovecii* antibodies in sera of patients with previous contact with *P. jirovecii*, which is supported by reports of high seropositivity for *P. jirovecii* in healthy individuals (Morris and Norris, 2012). Yet, the presence of these type of interactions does not impair the LFIA concept to be developed for two main reasons. The first one is based on the fact that these interactions will not be detected in the LFIA strip test because the search is directed to the presence of IgM anti-*P. jirovecii* antibodies, as this Ig class was the only one showing applicability in distinction of patients with active disease from not infected patients, with the ELISA results. The second reason is the consistent presence of a migration shift in the AGE assay resulting from different electrophoretic mobility’s of AuNP-RSA-Casein conjugates after interaction with the positive and negative samples (Figure 5). These shifts result from specific interactions between anti-*P. jirovecii* antibodies present in the sera of PcP patients and the RSA, which leads to a decrease in the migration of the AuNP-RSA-Casein conjugates after contact with the positive sample, functioning as a proof-of-concept for the LFIA to be developed. It’s important to notice that a more significant shift was achieved when using the Kex1 RSA. Although this was theoretically unexpected, since Kex1, unlike Msg, is not a specific or multicopy *P. jirovecii* surface antigen, it was consistent with our ELISA results that showed more significant differences between PcP and no PcP patient’s IgM levels with the Kex1 RSA than with the Msg RSA. Together, these results suggest that this antigen will provide better diagnostic performance to the LFIA test than the Msg RSA.

Even so, the next step was the assembly of the two LFIA strips for detection of IgM anti-*P. jirovecii* antibodies in human sera. For that, components from a commercial starter kit were tested. Based on the visual interpretation of the strip results and on the manufacturer’s recommendations for the development of LFIA with AuNP conjugates, the final components were selected. Then, the LFIA results were optimized to meet the following criteria: the appearance of color in the test and control lines within a reasonably short time (up to 10 min); presentation of a positive and negative result easily distinguished by the naked eye and confirmed by color quantification; minimum consumption of reagents for cost control. Taking this into consideration, pre-treatment steps were performed in the selected conjugate and sample pads. During the optimization process, it was noted that after adsorption, it was difficult to elute the AuNP-RSA-Casein conjugates from the conjugate pad (data not shown). Thus, as previously described by other authors that developed colloidal gold-based lateral-flow immunoassays (Kolosova et al., 2007; Li et al., 2015), we pre-treated the conjugate pad with a buffer containing 5% sucrose, 1% BSA and 0.5% Tween 20, to improve conjugates stability and re-solubilization. On the other hand, since the LFIA assay is only intended for the detection of IgM class antibodies and since AGE assays have shown that other immunoglobulins (probably IgG) can interact with our conjugates, we considered adding a preliminary step to eliminate some of the patient’s IgG antibodies before serum contact with the conjugates. For this purpose, a pre-treatment of the sample pad with anti-human IgG antibodies was made, as suggested by other authors which developed LFIA for detection of IgM antibodies (Li et al., 2015). The aim of this step was to increase the sensitivity of the test by decreasing the percentage of anti-*P. jirovecii* IgG antibodies that reach the conjugate pad, in order for the conjugates to be more available to interact with the target IgM anti-*P. jirovecii* antibodies.

The results from Figures 6A,C show that visually the test and control lines became easier to see with the naked eye and the results from Figures 6B,D confirmed this by color quantification. The comparative analysis of color intensity between strips before and after treatment, showed that the color intensity of both control and test lines has increased after treatment and that the software was not able to recognize the visible signal on the test line before treatment with the cut-off established. These results confirm that the pre-treatment steps improve the interpretation of the results, demonstrating that these steps are crucial to increase the assay sensitivity. On the other hand, the results showed a more intense color in the control line than in the test line. However, as the signal in the control line is suffering from drying effect (“coffee-ring”), we decided to make the following optimizations dispensing the control and test antibodies in a line instead of in a circle.

After, the sample dilution was also optimized (Figure 7). The intensity of the signals in the control and test lines increased with the dilution of the sample up to 1:50 in LFIA with the AuNP-Msg conjugates and up to 1:20 in LFIA with the AuNP-Kex1conjugates. Although signal weakening at higher dilutions was expected due to excessive sample dilution, the lack of test signals or the presence of a weaker signal at low dilutions, with both AuNP-RSA-Casein conjugates, was not expected. This is especially relevant for AuNP-Msg-Casein conjugates. We speculate that excessive levels of anti-*P. jirovecii* antibodies in the samples might lead to the formation of conjugate-antibody aggregates, preventing their free movement along the elution profile. The need for higher dilutions for conjugates with the Msg RSA than for conjugates with the Kex1 RSA was anticipated and consistent with our ELISA results, which demonstrated that serum levels of anti-Msg antibodies are higher than serum levels of anti-Kex1 antibodies. Taken together, these results demonstrate that sample dilution and sample pad pre-treatment are key factors that influence LFIA performance, and should be reevaluated when validating these LFIA in a large cohort study, before their implementation in the clinical practice.

Finally, triplicates of the optimized LFIA strips (see Supplementary Figure S15) were tested with a pool of serum specimens from patients with PcP (positive sample) and a pool of serum specimens from patients without *P. jirovecii* infection (negative sample) in the selected dilutions (Figure 8). During these assays, it was established that 3 min are enough for the sample to elute completely until the absorbing pad, giving a LFIA final result. The digital pictures (Figures 8A,C) and the color intensity analysis (Figures 8B,D) of the final results showed that in strips tested with the negative pool, only a colored line was visible on the control zone and detected by the color quantification software in all replicates. Additionally, in strips tested with the positive sample, a colored line was visible and detected by the software in both test and control zones as expected in all replicates, in both Msg and Kex1 LFIA strips.

However, these results also show that further optimization processes are needed. On the one hand, the variability between the assays (represented by the error bars in Figure 8), which may be justified by the lack of standardization of reagent application and strips assembly, should be minimized through automatic manufacturing processes. On the other hand, the results show that the color intensity in the control line is higher in strips tested with negative samples than in strips tested with positive samples. Although this could be explained by a higher number of free AuNP-RSA-Casein conjugates in strips tested with negative samples, it should be addressed in further optimizations by improving the conjugates:control antibodies ratio used. Finally, the results from Msg LFIA strips, showed that the conjugates seem to have formed aggregates. Although this did not impair the performance of the test, future optimization should focus on maintaining the stability of these conjugates after adsorption to the conjugate matrix, in order to obtain equally intense red lines in the LFIA strips.

## Conclusion

In conclusion, this study provides a proof-of-concept that a point of care diagnostic test for PcP can be developed and that both LFIA developed allow the detection of IgM anti-*P. jirovecii* reactive against both RSA. This is important because in our previous study (Tomás et al., 2016) and in this study, we verified that IgM levels could be used as a serological biomarker of *P. jirovecii* active infection. However, future work is needed in order to optimize and validate this diagnostic approach in a large prospective study with patients from different clinical groups, in order to assess the sensitivity, specificity and accuracy of the two LFIA strips proposed, individually and combined. Only with these results, it will be possible to confirm the need to study the reactivity against more than one RSA in parallel and then optimize a diagnostic kit, for implementation in the clinical practice. If both AuNP-RSA conjugates prove useful in PcP diagnosis, a multiplex strategy, based in the use of two conjugate pads for the simultaneous detection of two proteins (Zhu et al., 2013), could be adapted for the present LFIA strips.

The technology proposed in this study reveals to the scientific community how these less conventional conjugates can be used in the development of an alternative approach to the conventional diagnosis of PcP, reducing the need for the current invasive procedures used in the collection of respiratory specimens, as well as reducing time response and costs associated with PcP diagnosis. Ultimately, this study will help in the management of PcP in industrialized countries, also having a major impact on developing countries with low income and lack of technology, where PcP is an emerging disease with high prevalence and poorly controlled.

## Data Availability Statement

The raw data supporting the conclusions of this article will be made available by the authors, without undue reservation, to any qualified researcher.

## Ethics Statement

The studies involving human participants were reviewed and approved by the Instituto de Higiene e Medicina Tropical, Lisboa, Portugal. Written informed consent for participation was not required for this study in accordance with the national legislation and the institutional requirements. The animal study was reviewed and approved by the Instituto de Higiene e Medicina Tropical, Lisbon, Portugal.

## Author Contributions

AT, RF, and OM were responsible for the study design. AT wrote the manuscript. AT, MA, FC, and MP performed the experiments. OM, EP, and RF were responsible for reagents, materials, and analysis tools supplies. All authors contributed to the approval of the final version of the manuscript.

## Conflict of Interest

AT, FC, and OM have a patent PT109078 pending to Instituto de Higiene e Medicina Tropical (Lisbon, Portugal) related to the Msg RSA used in this study. The remaining authors declare that the research was conducted in the absence of any commercial or financial relationships that could be construed as a potential conflict of interest.
