# Supplementary material for: Development of a Gold Nanoparticle-Based Lateral-Flow Immunoassay for Pneumocystis Pneumonia Serological Diagnosis at Point-of-Care
Source: Front Microbiol. 2019 Dec 19;10:2917. doi: 10.3389/fmicb.2019.02917 (PMC6931265; doi:10.3389/fmicb.2019.02917)
Supplement: Supplementary file 1 [file Data_Sheet_1.pdf]

Supplementary Material

Supplementary Figures

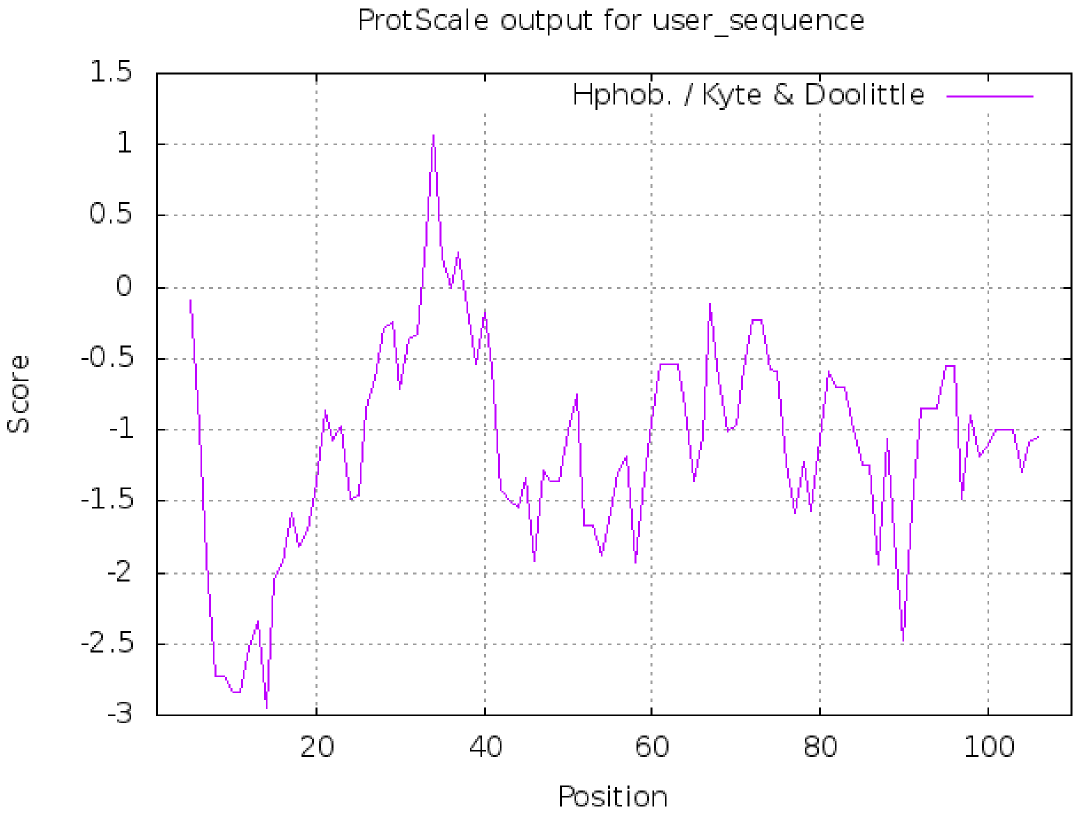

Supplementary Figure 1. Representation of hydrophilic behaviour of the Kex1 RSA according to Kyle & Doolittle hydropathic scale. The majority of the amino acid sequence of the RSA have a hydrophobic profile (score < 0), as was intended (ExPASy – ProtScale).

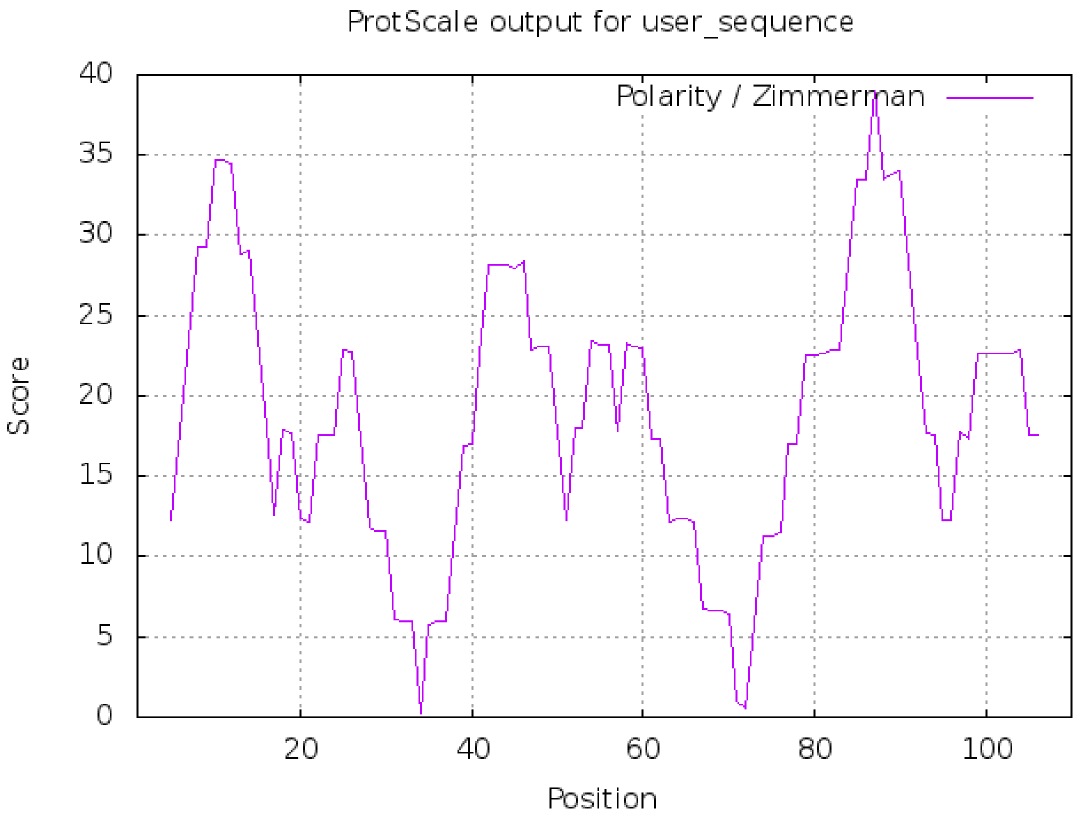

Supplementary Figure 2. Representation of polarity of the Kex1 RSA according to Zimmerman scale. The majority of the amino acid sequence of the RSA have a polar profile, as was intended (ExPASy – ProtScale).

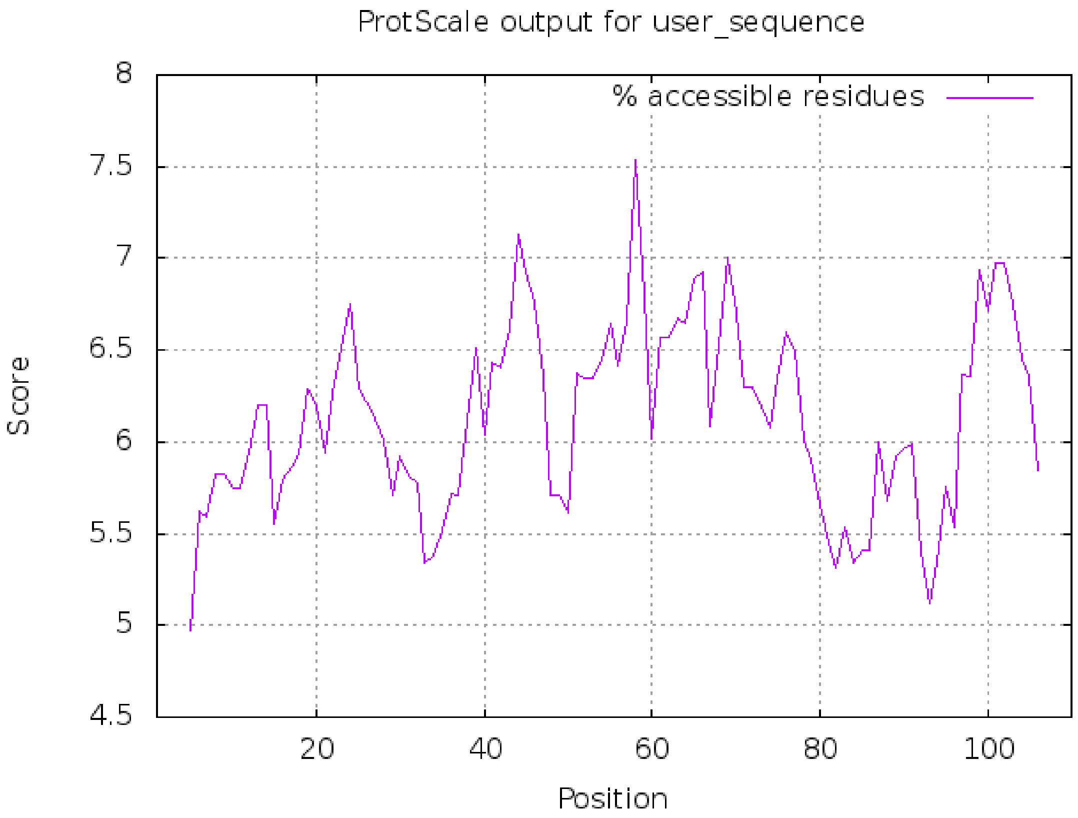

Supplementary Figure 3. Representation of accessibility of the residues of the Kex1 RSA. All the amino acid sequence of the RSA have an accessible profile, as was intended (ExPASy – ProtScale).

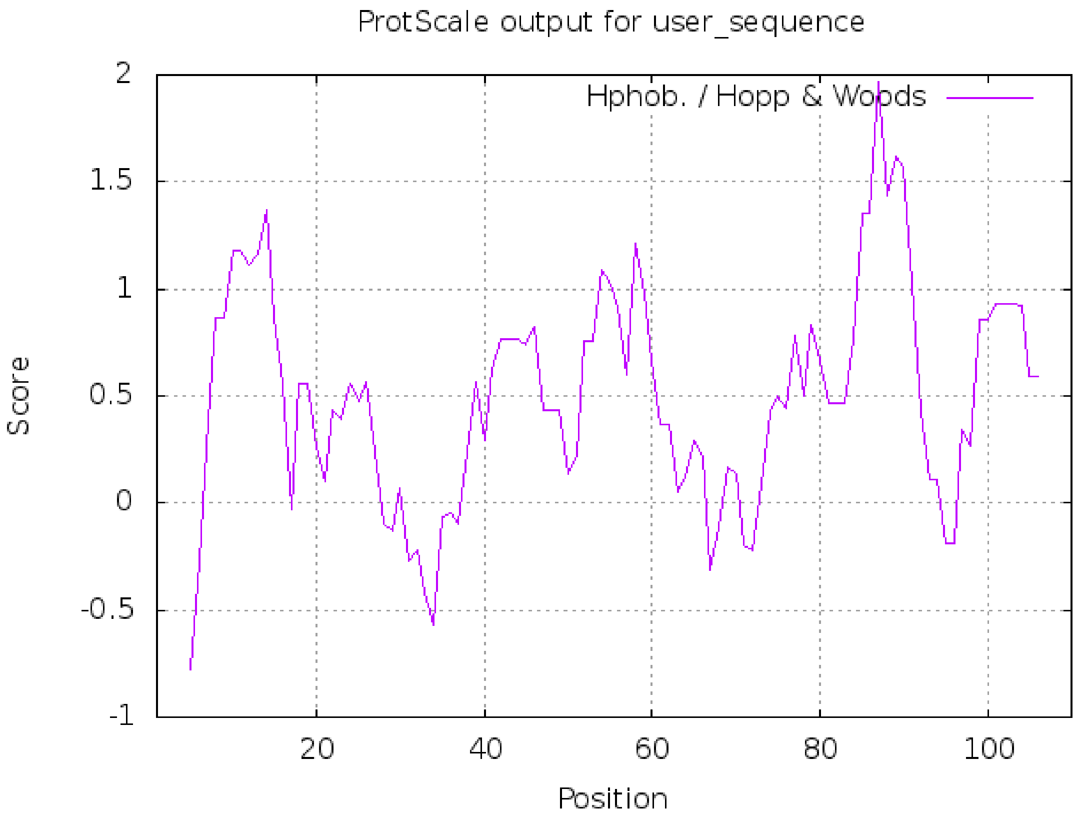

Supplementary Figure 4. Representation of antigenicity profile of the Kex1 RSA according to Hopp & Woods scale. The residues of the three epitopes selected have an antigenic profile while the glycine bridges have a less antigenic behaviour, as was intended (ExPASy – ProtScale).

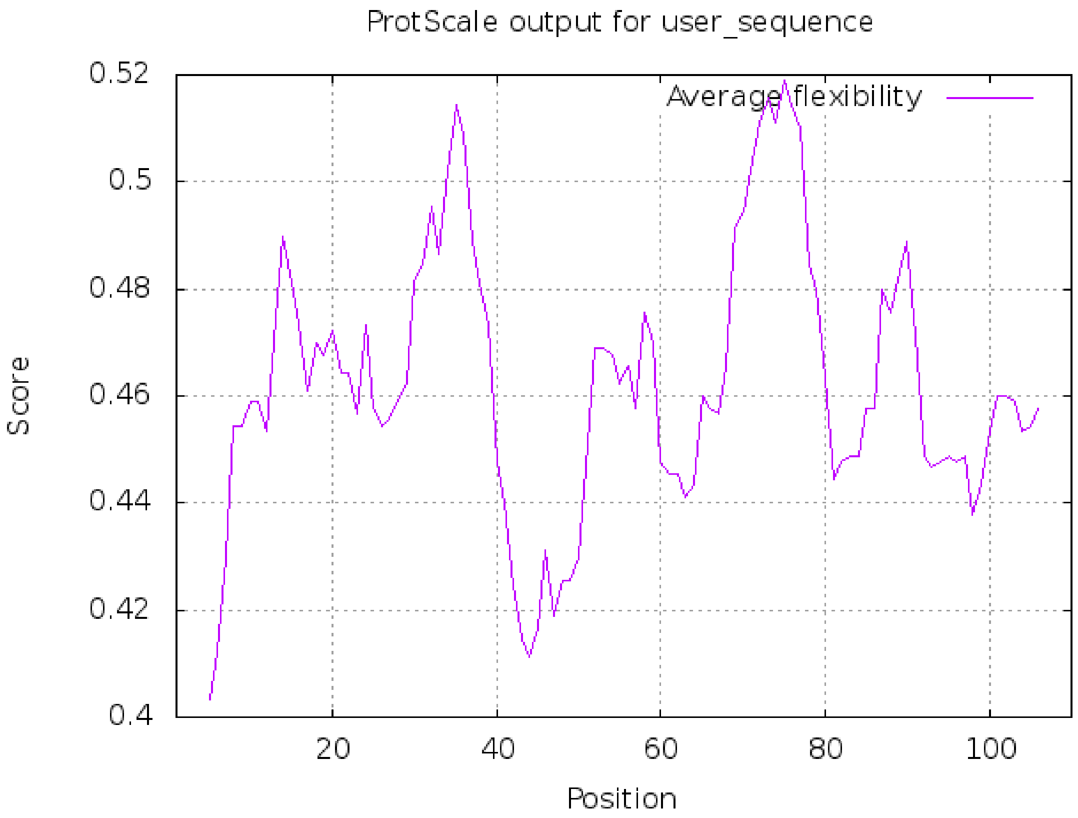

Supplementary Figure 5. Representation of the average flexibility profile of the residues of the Kex1 RSA. The residues have a positive average flexibility, as was intended (ExPASy – ProtScale).

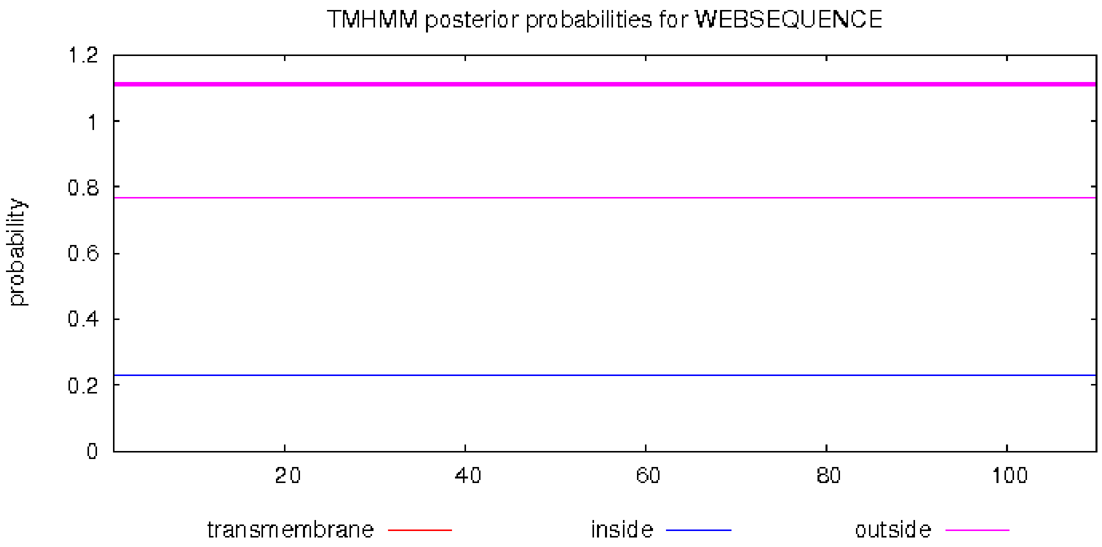

Supplementary Figure 6. Representation of the putative position on the cytoplasmic membrane of the amino acids present in the Kex1 RSA, through CBS – TMHMM – version 2.0 software. This figure pointed to a probability close to one for an external exposure over the whole region under consideration.

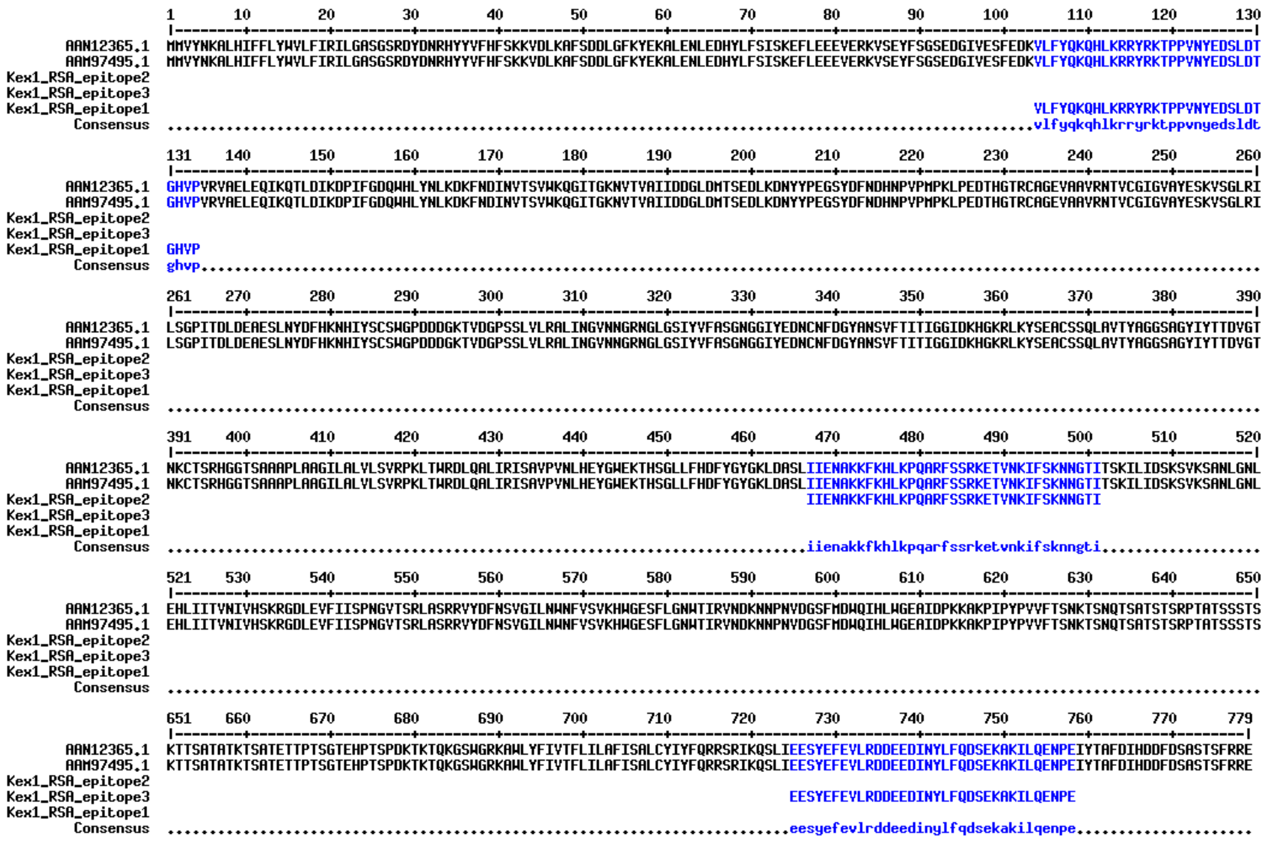

Supplementary Figure 7. Alignment of the amino acid sequences of the Kex1 protein from GenBank AAN12365.1 and AAM97495.1 and the three amino acid sequences of the regions selected to produce the final Kex1 RSA. This alignment shows that the Kex1 selected regions (region1: 104-134 aa; region 2: 467-501 aa; region 3: 725-758 aa) are located at conserved regions (Multalin version 5.4.1).

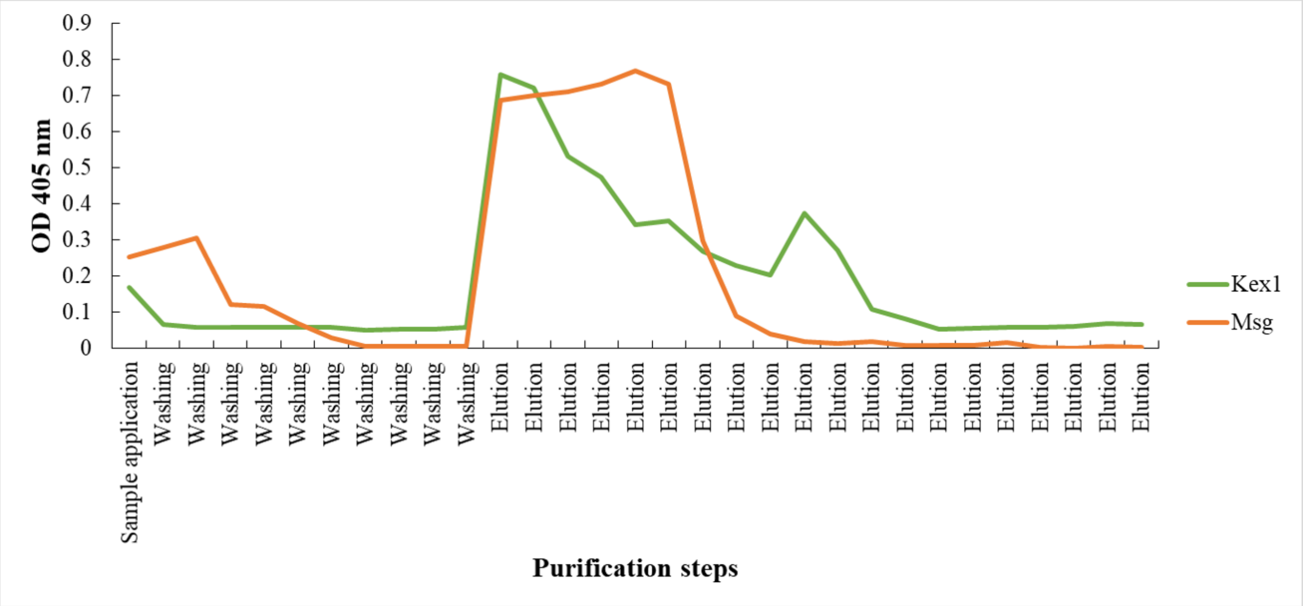

Supplementary Figure 8. Representation of indirect ELISA using anti-polyhistidine antibodies measurements (OD 405nm), throughout the purification process of Msg (orange) and Kex1 (green) RSA. Each purification step corresponds to 1 mL volume of sample, washing buffer and elution buffer, respectively.

(A)

(B)

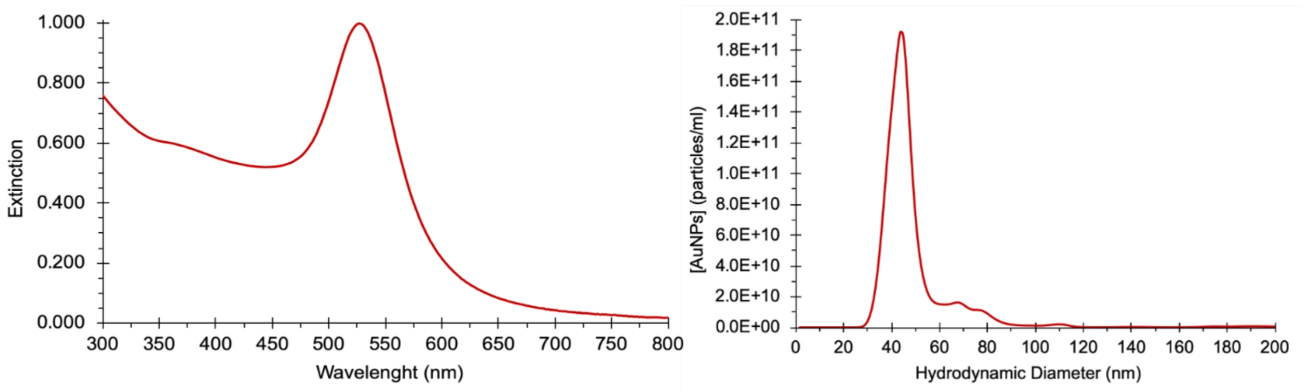

Supplementary Figure 9. AuNPs characterization through UV-Vis and NTA. **(A)** Visible spectrum of AuNPs functionalized with 11-MUA. **(B)** Hydrodynamic diameter distribution of the same functionalized AuNPs, obtained by nanoparticle tracking analysis.

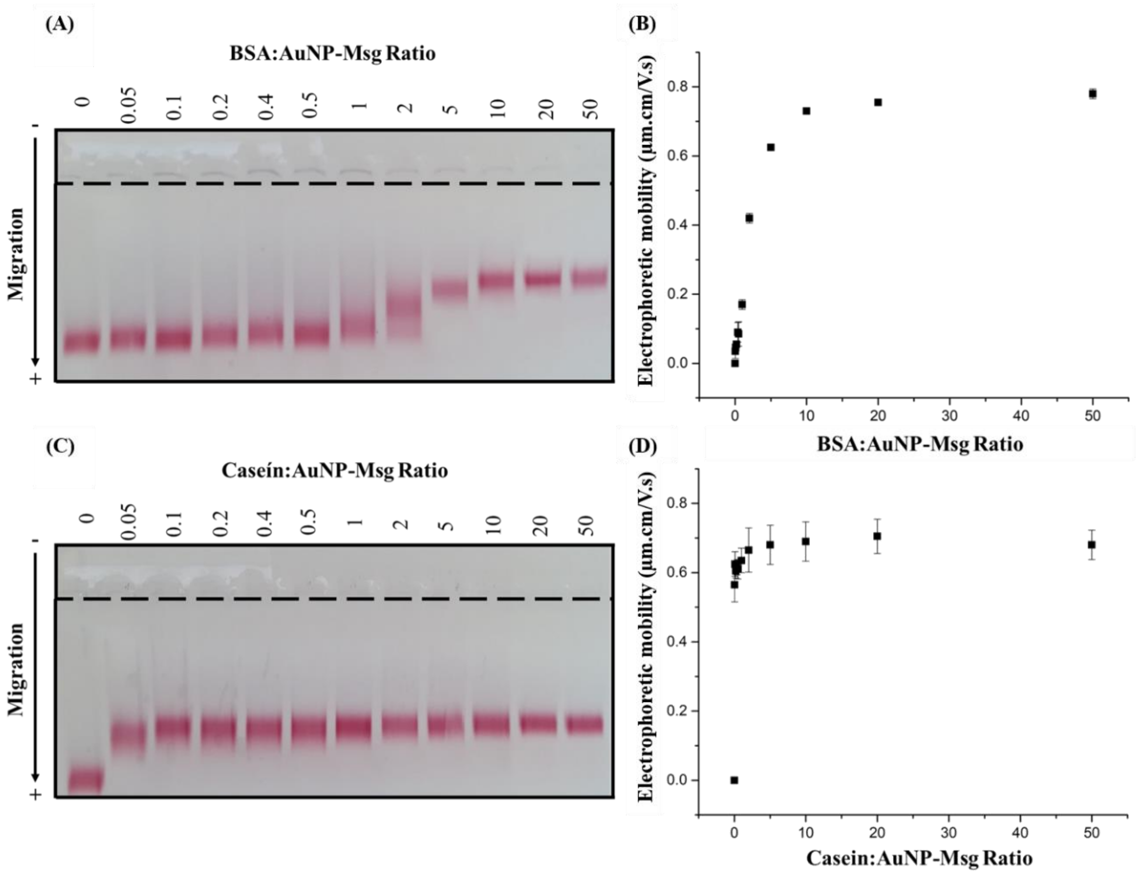

Supplementary Figure 10. AuNP-Msg-BSA and AuNP-Msg-Casein conjugates formation. Agarose gel electrophoresis at increasing ratios of BSA to AuNP-Msg **(A)** and Casein to AuNP-Msg **(C)**, showing the migration of each conjugate bands towards the positive electrode. Differences between the electrophoretic mobility of the AuNP-Msg conjugates and AuNP-Msg-BSA **(B)** or AuNP-Msg-Casein **(D)** conjugates at increasing ratios, calculated from the migration distances in gel. The eleven increased ratios (0.05 to 50) correspond to eleven increased protein concentration in nM: 11.7, 23.4, 46.9, 93.8, 117.2, 234.4, 468.8, 1172.1, 2344.2, 4688.4, 11720.9.

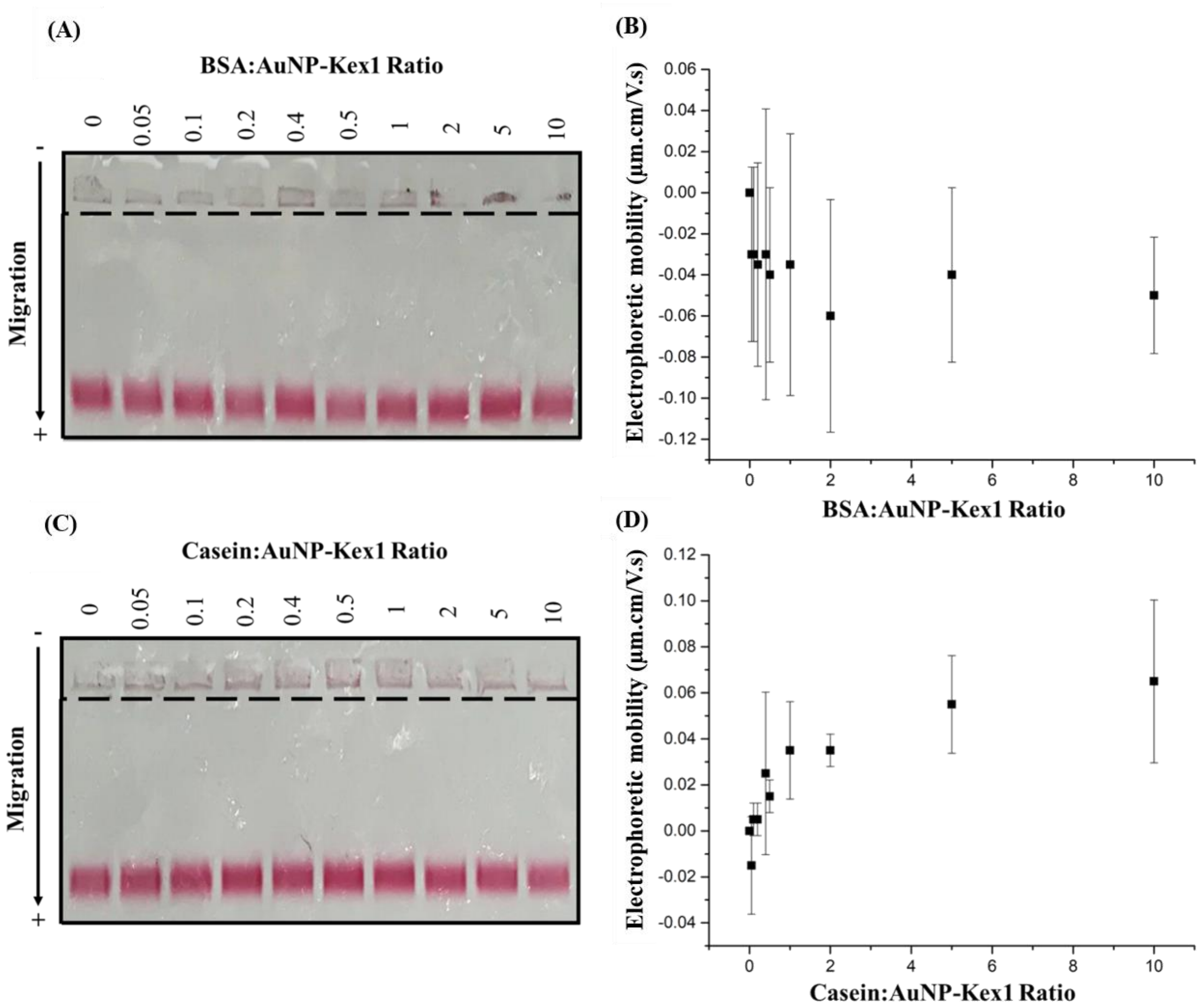

Supplementary Figure 11. AuNP-Kex1-BSA and AuNP-Kex1-Casein conjugates formation. Agarose gel electrophoresis at increasing ratios of BSA to AuNP-Kex1 **(A)** and Casein to AuNP-Kex1 **(C)**, showing the migration of each conjugate bands towards the positive electrode. Differences between the electrophoretic mobility of the AuNP-Kex1 conjugates and AuNP-Kex1-BSA **(B)** or AuNP-Kex1-Casein **(D)** conjugates at increasing ratios, calculated from the migration distances in gel. The nine increased ratios (0.05 to 50) correspond to nine increased protein concentration in nM: 11.7, 23.4, 46.9, 93.8, 117.2, 234.4, 468.8, 1172.1, 2344.2.

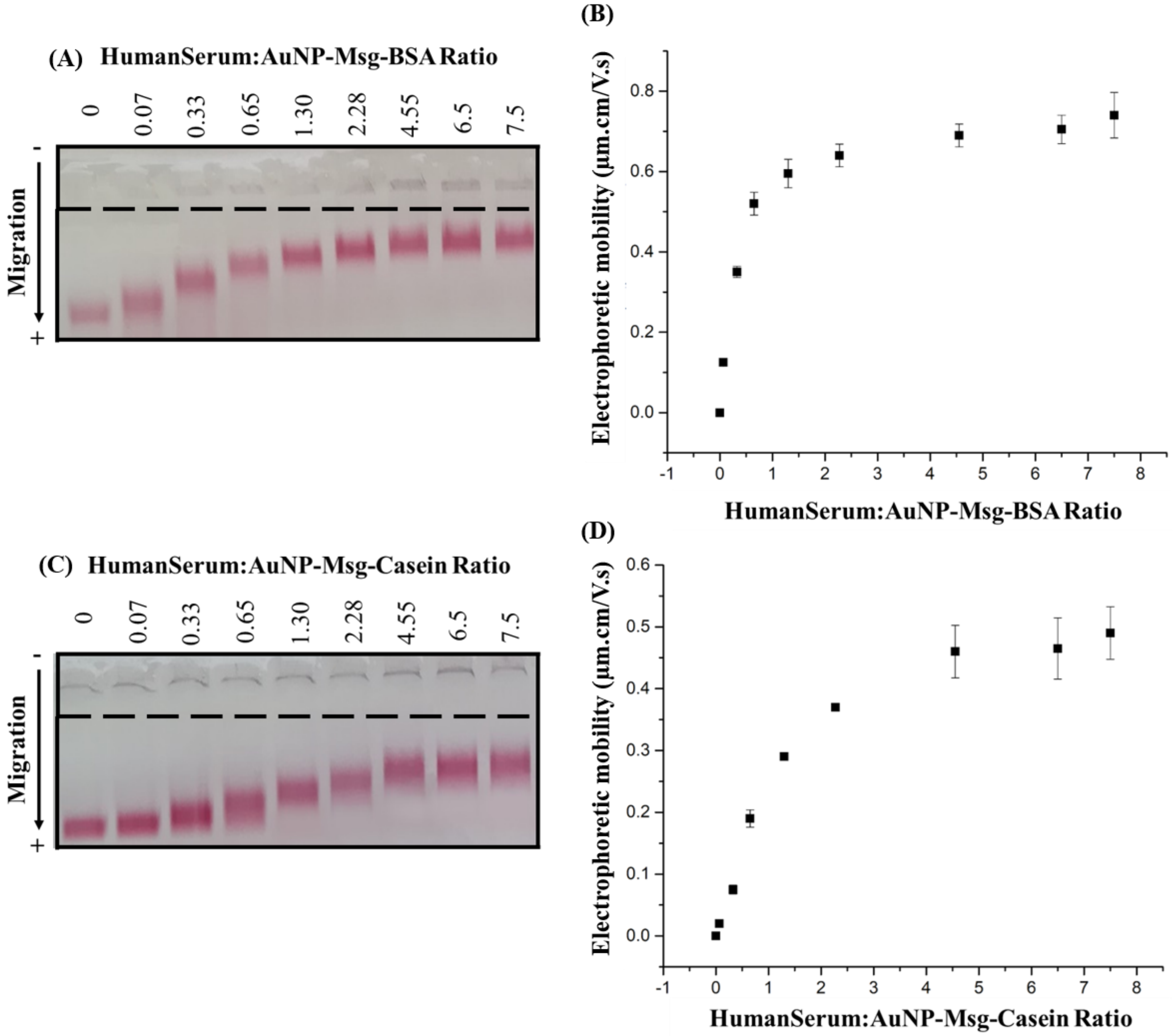

Supplementary Figure 12. AuNP-Msg-BSA-HumanSerum and AuNP-Msg-Casein-HumanSerum conjugates formation. Agarose gel electrophoresis at increasing ratios of human serum to AuNP-Msg-BSA **(A)** and AuNP-Msg-Casein **(C)**, showing the migration of each conjugate bands towards the positive electrode. Differences between the electrophoretic mobility of the blocked conjugates and AuNP-Msg-BSA-HumanSerum **(B)** or AuNP-Msg-Casein-HumanSerum **(D)** at increasing ratios, calculated from the migration distances in gel. The eight increased ratios (0.07 to 7.5) correspond to eight increased serum concentration in nM: 15.2, 76.2, 152.4, 304.7, 533.3, 1066.6, 1523.7, 1758.1.

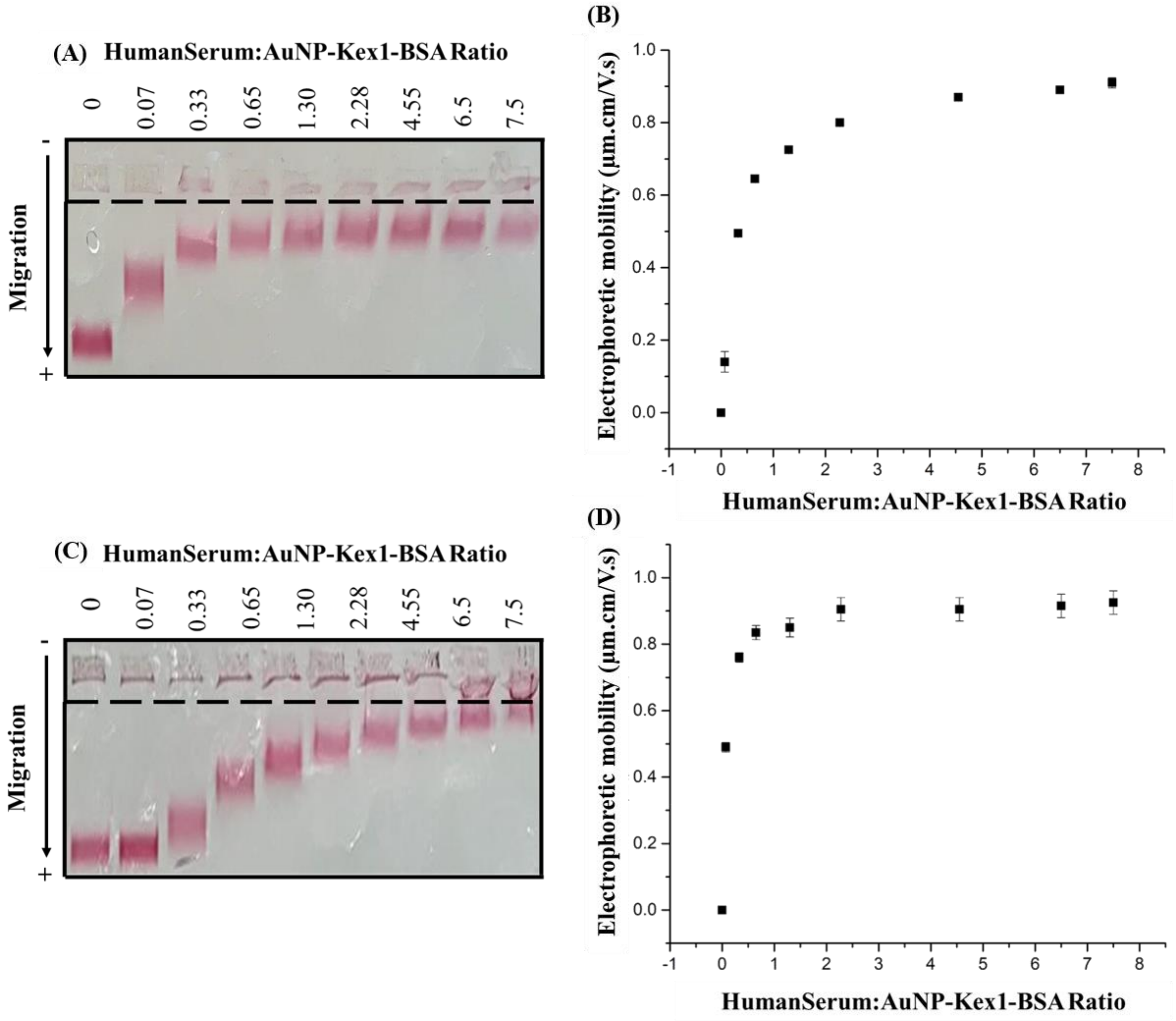

Supplementary Figure 13. AuNP-Kex1-BSA-HumanSerum and AuNP-Kex1-Casein-HumanSerum conjugates formation. Agarose gel electrophoresis at increasing ratios of human serum to AuNP-Kex1-BSA **(A)** and AuNP-Kex1-Casein **(C)**, showing the migration of each conjugate bands towards the positive electrode. Differences between the electrophoretic mobility of the blocked conjugates and AuNP-Kex1-BSA-HumanSerum **(B)** or AuNP-Kex1-Casein-HumanSerum **(D)** at increasing ratios, calculated from the migration distances in gel. The eight increased ratios (0.07 to 7.5) correspond to eight increased serum concentration in nM: 15.2, 76.2, 152.4, 304.7, 533.3, 1066.6, 1523.7, 1758.1.

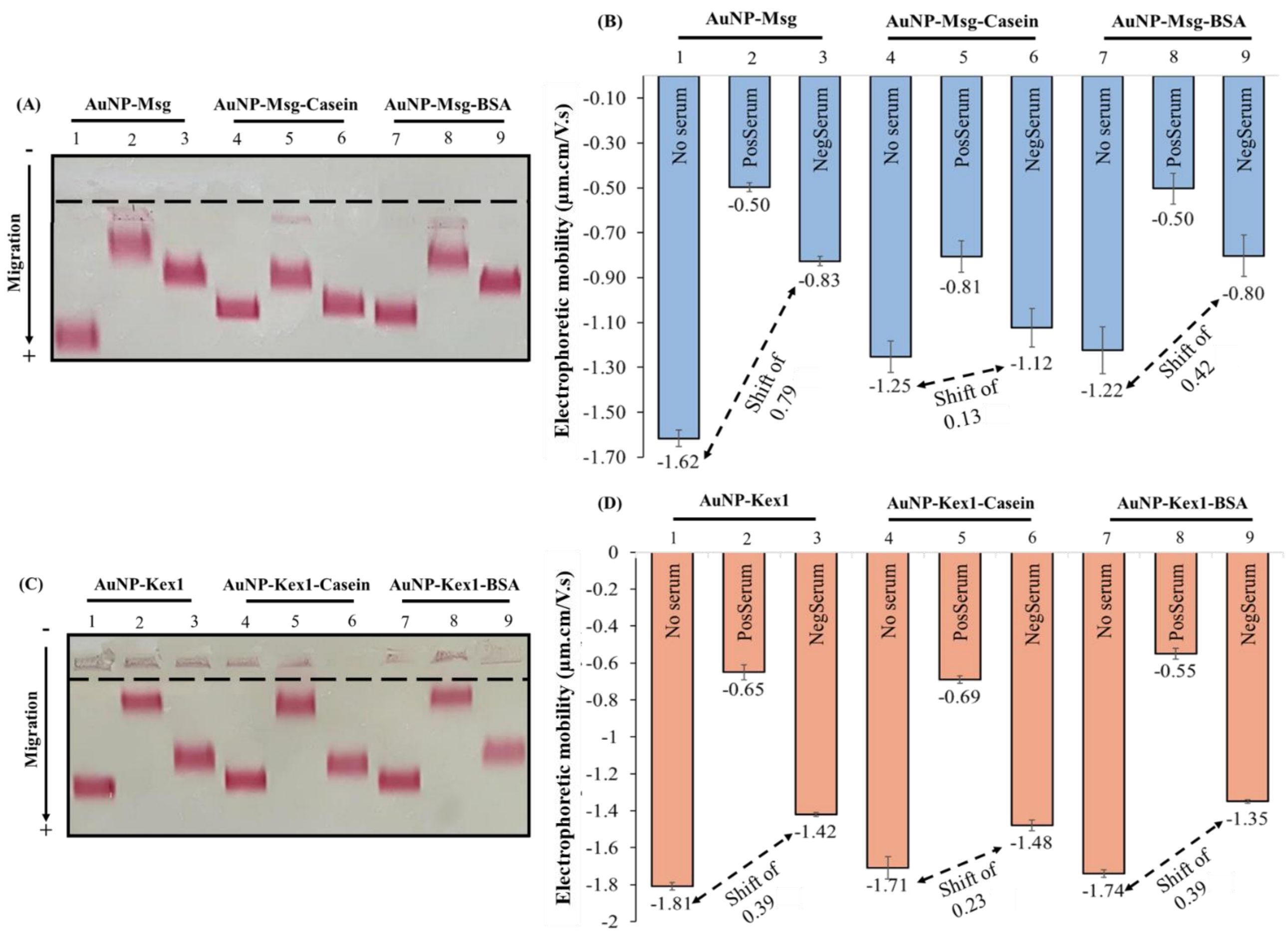

Supplementary Figure 14. Interaction of blocked and unblocked AuNP-Msg **(A, B)** and AuNP-Kex1 **(C, D)** conjugates with sera pools from patients with and without *P. jirovecii* infection. **(A, C)** Agarose gel electrophoresis of unblocked AuNP-RSA conjugates (1, 2, 3), casein blocked conjugates (4, 5, 6) and BSA blocked conjugates (7, 8, 9), before interaction with serum (1, 4, 7) and after interaction with positive (2, 5, 8) and negative (3, 6, 9) samples. **(B, D)** Electrophoretic mobility of the AuNP-RSA, AuNP-RSA-Casein and AuNP-RSA-BSA conjugates before interaction with human sera (No Serum) and after interaction with the positive sera pool (PosSerum) or the negative sera pool (NegSerum). Shifts (µm.cm/V.s) between AuNP-RSA and negative sera are indicated, evidencing the effect of blocking. Standard deviation bars correspond to triplicate experiments.

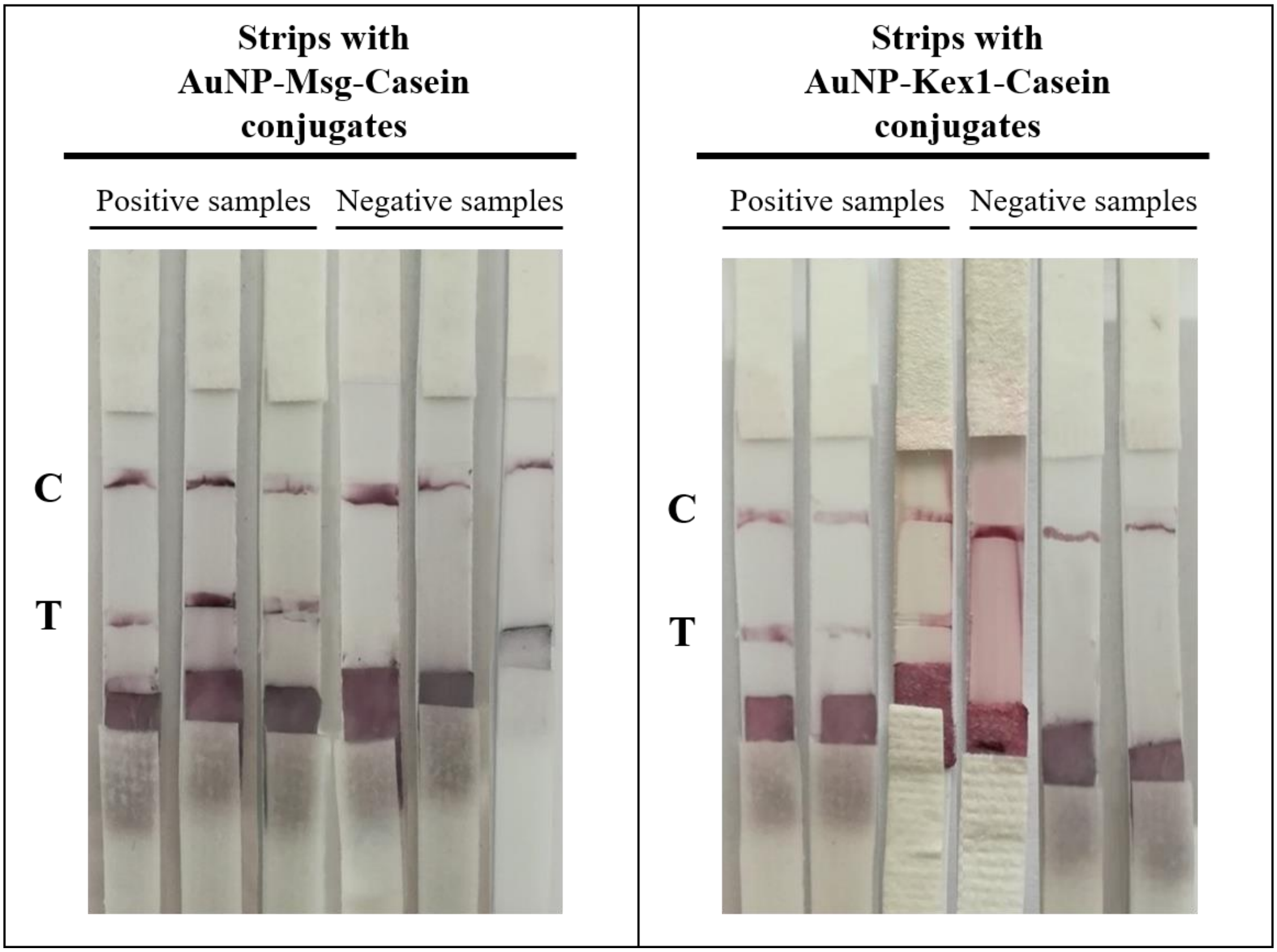

Supplementary Figure 15. Digital pictures of the triplicate experiments of LFIA strips containing AuNP-Msg-Casein conjugates and AuNP-Kex1-Casein conjugates, tested with sera from patients with (positive samples) and without (negative samples) PcP.
